# A nuclear enterprise: zooming in on nuclear organization and gene expression control in the African trypanosome

**DOI:** 10.1017/S0031182020002437

**Published:** 2021-09

**Authors:** Joana R. C. Faria

**Affiliations:** The Wellcome Trust Centre for Anti-Infectives Research, School of Life Sciences, University of Dundee, Dow Street, Dundee DD1 5EH, UK

**Keywords:** Antigenic variation, genome architecture, nuclear bodies, RNA processing, gene expression, transcription factories, *Trypanosoma brucei*, Trypanosomatids, VSG

## Abstract

African trypanosomes are early divergent protozoan parasites responsible for high mortality and morbidity as well as a great economic burden among the world's poorest populations. Trypanosomes undergo antigenic variation in their mammalian hosts, a highly sophisticated immune evasion mechanism. Their nuclear organization and mechanisms for gene expression control present several conventional features but also a number of striking differences to the mammalian counterparts. Some of these unorthodox characteristics, such as lack of controlled transcription initiation or enhancer sequences, render their monogenic antigen transcription, which is critical for successful antigenic variation, even more enigmatic. Recent technological developments have advanced our understanding of nuclear organization and gene expression control in trypanosomes, opening novel research avenues. This review is focused on *Trypanosoma brucei* nuclear organization and how it impacts gene expression, with an emphasis on antigen expression. It highlights several dedicated sub-nuclear bodies that compartmentalize specific functions, whilst outlining similarities and differences to more complex eukaryotes. Notably, understanding the mechanisms underpinning antigen as well as general gene expression control is of great importance, as it might help designing effective control strategies against these organisms.

## Introduction

Trypanosomes are members of the Euglenozoa, a group of organisms within the *Incertae sedis Eukarya* (ex-Excavata) supergroup (Adl *et al*., 2019). These organisms are likely to have branched very early during evolution, which may explain the vast number of unorthodox features that define their biology (Navarro *et al*., 2007; Adl *et al*., 2019). Euglenozoa include Euglenids, Phytomonads and Trypanosomatids, free-living phagotrophs, plant and animal parasites, respectively (Adl *et al*., 2019). Trypanosomatids include several parasitic protozoa that cause a huge health and economic burden amongst the world's poorest populations; these include *Leishmania* sp, *Trypanosoma cruzi*, *Trypanosoma brucei*, *Trypanosoma congolense* and *Trypanosoma vivax*. Notably, climate change, increased mobility and mass migration pose great challenges to our ability to control diseases caused by these organisms, rendering the need for new drugs to fight new parasite strains and resistance emergence imperative. Therefore, a detailed molecular understanding of fundamental aspects of their cell biology, gene expression, metabolism and interaction with the hosts is critical to design effective control strategies.

*Trypanosoma brucei* is the causative agent of sleeping sickness and nagana in humans and cattle, respectively, and has been used for decades as a model organism for this group mostly given its genetic tractability and available tools for reverse and forward genetics (Djikeng *et al*., 2001; Alsford *et al*., 2011; Dean *et al*., 2017; Rico *et al*., 2018).

*Trypanosoma brucei* is transmitted through the bite of a tsetse fly and rapidly differentiates into ‘slender’ bloodstream forms (BSFs) in the mammalian host. The slender forms are capable of sensing the population density, which triggers differentiation into stumpy forms. The latter are pre-adapted to life in the tsetse, where they will eventually differentiate into the procyclic forms. In the mammalian host, besides the BSFs, these parasites can occupy multiple tissues (brain, adipose tissue, skin, etc.), some recently identified as important reservoirs (Capewell *et al*., 2016; Trindade *et al*., 2016).

**Fig. 1.**
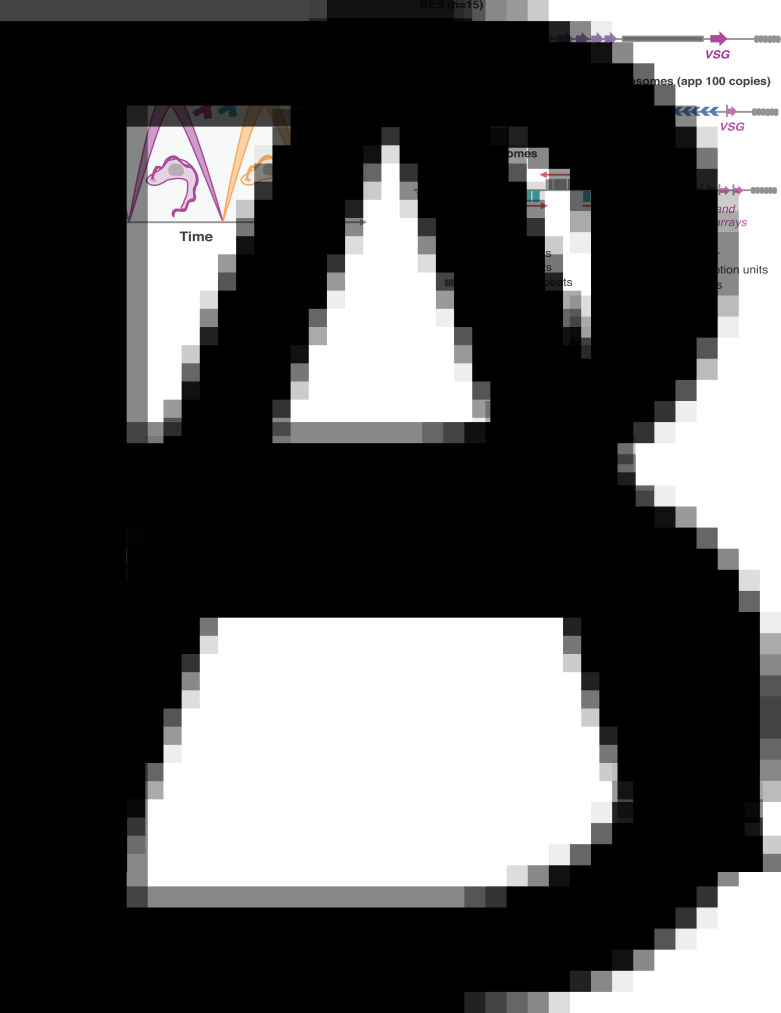
Antigenic variation in *T. brucei* bloodstream forms. (A) Antigenic variation. Waves of parasitaemia are a hallmark of infections by African trypanosomes in mammals. This is due to waves of parasites expressing different VSG coats (different colours). VSGs are highly immunogenic, typically triggering an effective and lasting immune response (immunosuppression can occur later during infection). This illustration is a simplified depiction of the *in vivo* dynamics, indeed, at any time point the populations can be much more complex than represented: these may include large numbers of different clonal VSG variants. (B) Genomic organization of *VSG* genes. Bloodstream *VSG* expression-sites (BESs) contain expression-site-associated genes (*ESAGs*), which are located between the promoter and the 70 bp repeats. The *VSG* genes are near telomeric repeats. Large extensions of 50 bp repeats are located upstream of all BESs. Metacyclic *VSG* expression-sites (MESs) lack *ESAGs* and are expressed in metacyclic trypomastigotes in the salivary glands of the tsetse fly. Pol-II transcribed genes are organized in long polycistronic transcription units in the 11 megabase (Mb) size chromosomes. The arrows indicate the direction of Pol-II transcription. *VSG* genes or pseudogenes are organized in sub-telomeric regions of megabase chromosomes or at the telomeres of minichromosomes.

Mammalian-infective *T. brucei* undergoes antigenic variation to successfully evade the host adaptive immune responses (Fig. 1A), similarly to other pathogens such as malaria and giardiasis causing parasites (Duraisingh and Horn, 2016). For that purpose, it relies on a vast genetic repertoire of genes that encode for their variant surface glycoprotein (>2500 *VSG* genes and pseudogenes), approximately one-third of its genome (Berriman *et al*., 2005; Muller *et al*., 2018). There are two key features for successful antigenic variation: (1) the ability to express a single antigen from myriad possibilities (monogenic expression); (2) the ability to switch from one antigen isoform to another (Duraisingh and Horn, 2016). However, despite the vast genetic repertoire, a *VSG* gene can only be expressed from a limited subset of sub-telomeric transcription units known as expression-sites (ESs) (Navarro and Cross, 1996; Hertz-Fowler *et al*., 2008; Fig. 1B). *VSG*-ESs are polycistronic transcription units (PTUs) that share the same DNA elements, and yet, one is *active* whereas the remaining are *silent* – a classic epigenetic paradigm (Duraisingh and Horn, 2016).

The molecular understanding of the mechanisms underpinning antigenic variation is critical as it sustains persistent infections and has greatly challenged vaccine development against these organisms. This review will be focused on nuclear compartmentalization and how it affects or might affect both antigen and global gene expression in the African trypanosome. Overall, *T. brucei* nuclear architecture and mechanisms for gene expression control follow some of the classic conventions but also present phenomenal dissimilarities when compared to so-called *model eukaryotes*.

## Genome organization

Eukaryotic genomes are condensed by several orders of magnitude; such compaction is critical to fit into the nucleus of a cell. This is achieved by coiling the DNA around histones forming chromatin fibres, which are subsequently arranged into more complex high-order structures such as loops, domains and compartments (Gibcus and Dekker, 2013; Finn and Misteli, 2019). Several of these architectural features are conserved across the evolutionary tree, suggesting an elementary role of spatial organization in genome function and gene expression control (Foster and Bridger, 2005). Indeed, DNA spatial organization and compartmentalization has been found to play a key role in the regulation of gene expression and recombination in multiple organisms. In mammals on a larger scale, two major sub-nuclear compartments can be defined, one is transcription-permissive (compartment A) and the other transcription-repressive (compartment B), roughly corresponding to euchromatin and heterochromatin, respectively (Gibcus and Dekker, 2013; Finn and Misteli, 2019). Further, within chromatin domains known as topologically associating domains (TADs), chromatin loops modulate interactions between promoters and distal regulatory elements, ultimately impacting gene expression (Rao *et al*., 2014; Schoenfelder and Fraser, 2019). TADs are usually defined by boundary elements containing architectural chromatin proteins; these include cohesin, CCCTC-binding factor (CTCF) and histone variants (Millau and Gaudreau, 2011; Merkenschlager and Odom, 2013).

### The core genome of the African trypanosome

*Trypanosoma brucei* has a diploid genome, the haploid nuclear genome (32 Mbp) is divided into three classes of linear chromosomes: 11 pairs of megabase chromosomes (at least 1 Mbp), one to five intermediate-sized chromosomes and more than 100 minichromosomes (50–150 kbp) (Wickstead *et al*., 2004; Berriman *et al*., 2005). The megabase chromosomes contain all RNA-Polymerase-II (Pol-II) transcribed genes and *VSG*-ESs (Berriman *et al*., 2005; Muller *et al*., 2018). The minichromosomes are highly repetitive and many also contain *VSG* genes (Wickstead *et al*., 2004; Fig. 1B). Additionally, *T. brucei* has an unknown number of highly repetitive circular extra-chromosomal DNAs of unknown function (Alsford *et al*., 2003).

In trypanosomes, electron-dense chromatin regions can be found close to the nuclear periphery and their arrangement is developmentally regulated (Belli, 2000; Elias *et al*., 2001; Navarro *et al*., 2007). Indeed, in *T. brucei*, chromosome conformational capture (Hi-C) revealed that the transcribed chromosome core regions and the sub-telomeric regions coding for the large reservoir of silent *VSG* genes appear to fold into structurally distinct compartments (Muller *et al*., 2018), similar to active A and silent B compartments described in mammalian cells (Schoenfelder and Fraser, 2019). Further, the relative interaction frequency was substantially higher across sub-telomeric regions compared to core regions, indicating that sub-telomeres are more compact than the core region. Additionally, centromeres and junctions between the core and sub-telomeres were found to be the most prominent boundaries of DNA compartments (Muller *et al*., 2018).

Regarding architectural chromatin proteins, while CTCF appears to be absent in non-metazoans (Heger *et al*., 2012), the major subunit of cohesin is present in *T. brucei* and its depletion is lethal (Landeira *et al*., 2009). Moreover, histone variants (*H3V* and *H4V*) also function as architectural proteins in this organism (Muller *et al*., 2018). Indeed, studies in *T. brucei H3V* and *H4V* knockout cell lines revealed changes in global genome architecture and local chromatin configuration, which triggered switches in *VSG* expression (Muller *et al*., 2018).

### The telomeres and sub-telomeres

Genome sequences of *T. brucei* and *Plasmodium* revealed that Pol-II transcribed genes are located in the central core and antigen genes are located in sub-telomeric regions (Berriman *et al*., 2005; Otto *et al*., 2018; Fig. 1B).

The telomere is a special functional complex at the end of linear chromosomes, consisting of tandem repeat DNA sequences and associated proteins, which can form a specialized heterochromatic structure that suppresses the expression of genes located at the sub-telomere, known as telomere position effect or telomeric silencing (Ottaviani *et al*., 2008). Telomeres are essential for genome integrity and chromosome stability in eukaryotes and their synthesis is mainly achieved by the cellular reverse transcriptase telomerase, an RNA-dependent DNA polymerase that adds telomeric DNA to telomeres (Cong *et al*., 2002). Telomerase activity was found to be absent in most normal human somatic cells, which is intimately related with the ageing process, but present in over 90% of cancerous cells (Cong *et al*., 2002). Notably, telomere-binding proteins play critical roles on the maintenance of telomere length, telomere heterochromatin formation, regulation of the telomeric transcript levels, among others (Ottaviani *et al*., 2008). The mammalian telomere complex has been well characterized and contains six core proteins that include TRF1, TRF2, TIN2, RAP1, TPP1 and POT1 (de Lange, 2005). Additionally, an integral component of telomeric heterochromatin is the telomeric repeat-containing RNA (TERRA), a large non-coding RNA whose transcription occurs at most or all chromosome ends. Further, R-Loops have been identified at the telomeres, these are three-stranded nucleic acid structures that contain a DNA:RNA hybrid. R-Loops can play an important role in a number of cellular functions but they can also be an instability factor (Tan and Lan, 2020).

In the insect-stage, trypanosome telomeres tend to be close to the nuclear periphery, but this is much less pronounced in the mammalian-stage (DuBois *et al*., 2012). In *T. brucei*, besides the telomerase components (Dreesen *et al*., 2005; Sandhu *et al*., 2013), which are critical for telomere maintenance, several other telomere proteins have been identified. Among these, *Tb*TRF, a functional homologue of mammalian TRF2, a *Tb*TRF-interacting factor, TIF2, RAP1 and TelAP1 (Yang *et al*., 2009; Jehi *et al*., 2014*a*, 2014*b*; Reis *et al*., 2018). Except for TelAP1, all the other factors are essential for cell viability; *Tb*TRF and *Tb*TIF2 are critical for telomere integrity and their depletion leads to an increase in double-strand breaks and increased *VSG* switching (Jehi *et al*., 2014*a*, 2014*b*). *Tb*RAP1 interacts with *Tb*TRF and its depletion leads to derepression of silent *VSG*-ESs in the mammalian-infective stage, but also in insect-stage cells, where *VSG* expression is developmentally shut down (Yang *et al*., 2009). Further, *Tb*RAP1-mediated silencing has a stronger impact on telomere proximal genes (Yang *et al*., 2009). Moreover, by associating with telomere chromatin, *Tb*RAP1 also suppresses the expression of the TERRA transcripts and telomeric R-Loops, consistent with a role on telomere integrity (Nanavaty *et al*., 2017). Recent studies on *T. brucei* ribonuclease H enzymes, endonuclease enzymes that catalyse the cleavage of RNA in an RNA/DNA substrate, also showed that R-loops at the telomere and the sub-telomere affect *VSG* switching frequencies (Briggs *et al*., 2018, 2019).

Interestingly, the nuclear phosphatidylinositol 5-phosphatase (PIP5Pase), part of the inositol phosphate pathway, has been recently shown to interact with *Tb*RAP1 in a ~0.9-MDa complex (Cestari *et al*., 2019). The inositol phosphate pathway regulates several cellular processes in eukaryotes including chromatin remodelling and gene expression, and had been shown to have a role on telomere silencing and *VSG* monogenic expression in *T. brucei* (Cestari and Stuart, 2015).

In summary, in *T. brucei* (similarly to *Plasmodium*), Pol-II transcribed genes are located in the central core whereas the antigen genes are located in sub-telomeric regions (Berriman *et al*., 2005; Otto *et al*., 2018). This chromosome partitioning may be important to fine-tune recombination in regions that encode for antigens and to ensure that all but one antigen is repressed. Similarly to *Plasmodium*, there is a large amount of evidence that supports a role for telomeric chromatin in *VSG* gene silencing (Duraisingh and Horn, 2016). Moreover, the sub-telomeric location of *VSG*-ESs is thought to favour recombination, since these sites are rather unstable (Glover *et al*., 2013). Recombination-based and transcriptional mechanisms can lead to *VSG* switching, but undoubtedly recombination makes the largest contribution in *T. brucei* compared to *Plasmodium*. Indeed, telomere integrity and stability impacts *VSG* switching frequencies and has been also proven critical to maintain *VSG* monogenic expression (reviewed by Saha *et al*., 2020). Notably, one of the many remaining outstanding questions is how the active *VSG*-ES escapes telomeric silencing.

Remarkably, the active *VSG*-ES and the silent *VSG*-ESs reside within distinct nuclear compartments; the importance of nuclear compartmentalization on global gene expression control and *VSG* expression, in particular, will be addressed in the next chapter.

## Nuclear compartmentalization

The nucleus is a double lipid bilayer enclosed organelle, which separates genomic DNA from the rest of the cell. Its architecture shields the genome from the sources of damage whilst providing opportunities for gene expression regulation (reviewed by Lin and Hoelz, 2019). There is ample evidence in multiple eukaryotes that the transcriptional activity of genes is influenced by nuclear organization, which changes during differentiation and development. Indeed, the regulated expression of genes during development is influenced by the availability of regulatory proteins and the accessibility of the DNA to the transcriptional machinery (Finn and Misteli, 2019). In eukaryotes, heterochromatin, which is highly compact, is mainly located at the nuclear periphery, whereas the less compact euchromatin occupies a more interior nuclear position.

Additionally, key nuclear functions such as transcription, replication or RNA processing are not homogeneously distributed throughout the nucleus and can be compartmentalized. Such compartmentalization within the nucleoplasm enables functional specialization, separation of conflicting processes as well as increasing the concentration of specific factors at their target point of action (Finn and Misteli, 2019).

Two main models of nuclear organization emerged in the past. A deterministic model proposed that specific structural elements in the nucleus assembled into a scaffold that was then used by transcriptional processes, resulting in transcriptional compartmentalization, which was independent of active processes. Chromosome position would therefore be maintained by interactions with the scaffold (Misteli, 2007). In striking contrast, in a self-organization model, functional sites were formed depending on the gene activation status and without the need for predefined structures; chromosome position would therefore be established by chromatin itself and interactions with functional sites. Arguably, experimental data from many model systems strongly favour self-organization models over deterministic models. For instance, perturbing nuclear lamins, one of the prime structural components of the nucleus, has a modest impact on the spatial organization of transcription and pre-mRNA splicing sites, arguing against deterministic models (Spann *et al*., 1997). Conversely, perturbation of most active nuclear processes results in rapid chromatin architectural changes, consistent with self-organization models (Misteli, 2007).

### The nuclear periphery

At the nuclear periphery, there is a meshwork, designated nuclear lamina (NL), which in mammals is composed mainly by nuclear lamins. A growing number of nuclear proteins are known to bind lamins and are implicated in nuclear and chromatin organization, mechanical and genome stability, cell signalling, gene regulation, among others (Dechat *et al*., 2008). Notably, many molecules must be able to traffic between the nucleus and the cytoplasm, rendering nucleo-cytoplasmic transport absolutely critical for cell survival. The trafficking of macromolecules in and out of the nucleus occurs through nuclear pore complexes (NPCs) (reviewed by Lin and Hoelz, 2019).

#### Nuclear pore

NPCs are massive macromolecular assemblies! In humans, each NPC consists of ~1000 protein subunits, designated nucleoporins, rendering it one of the largest protein complexes in nature (~110 MDa). Each NPC is located in and stabilizes an ~800 Å-wide nuclear pore, which is generated by the fusion between the inner and outer nuclear membranes (reviewed by Lin and Hoelz, 2019).

NPCs are critical to maintain the nuclear integrity by preventing macromolecules from freely diffusing in or out of the nucleus. Macromolecules smaller than ~40 kDa can passively diffuse through the diffusion barrier, whereas larger macromolecules generally do not. Facilitated transport through NPCs is rapid, adding up to hundreds to thousands of macromolecules per second. Notably, NPCs conduct their cargos in their native state, allowing macromolecules to act immediately after transport, for instance during signal transduction (reviewed by Lin and Hoelz, 2019).

Most NPC proteins typically form a symmetric core that possesses an 8-fold rotational symmetry (nucleoporins are incorporated in multiples of eight). This symmetric core surrounds the central transport channel and functions as the scaffold onto which asymmetric nucleoporins attach on the cytoplasmic and nuclear compartments to form structures known as the cytoplasmic filaments and nuclear basket, respectively (reviewed by Lin and Hoelz, 2019). One inner ring that is embedded within the nuclear envelope, and two outer rings that reside on the inner or outer nuclear membrane generate the symmetric core itself. The major constituent of the outer rings in the NPC is the coat nucleoporin complex, which serves as a structural scaffold and docking site for other nucleoporins. The nuclear basket, composed of Nup153, Nup50 and Tpr, also serves as a hub for organising nuclear architecture and modulating gene transcription, mRNA processing and export (reviewed by Lin and Hoelz, 2019).

The majority of NPC architecture appears to be conserved throughout the Eukaryota and was already established in the last common eukaryotic ancestor (DeGrasse *et al*., 2009). However, although the proteins and complexes are rather conserved, their arrangements can differ substantially between cells in the same organism or even within the same cell type at the single cell level (Ori *et al*., 2013). Specifically, how the NPC connects with the lamina and mRNA transport is likely to be highly divergent between different lineages (Rout *et al*., 2017).

**Fig. 2.**
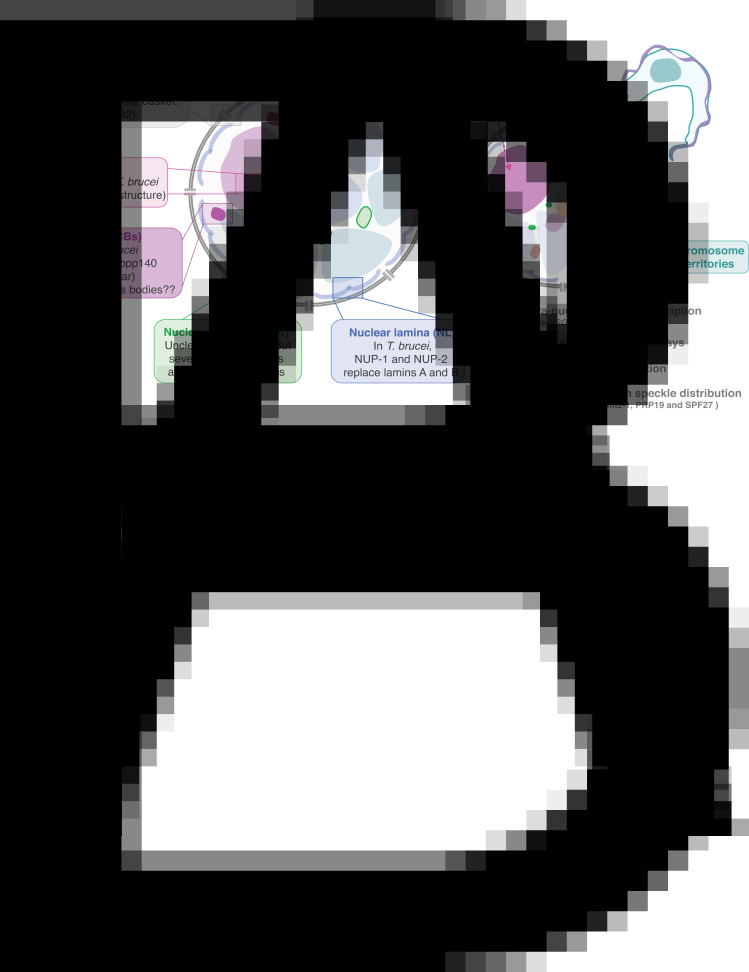
Nuclear organization and nuclear bodies. (A) The schematics represents the mammalian nucleus, the lateral boxes highlight differences in *T. brucei*. (B) The schematics represents the nuclear organization and nuclear bodies in *T. brucei*. Trypanosome-specific compartments are highlighted, such as the bloodstream form (BSF)-specific expression-site body (active-*VSG*-Expression-Site transcription; extra-nucleolar Pol-I transcription) and the *Spliced Leader* (*SL*)-array transcription compartments.

Proteomics analyses of NPC-containing fractions from *T. brucei* provided a comprehensive inventory of its nucleoporins, which clearly share a similar fold type, domain organization, composition and modularity in comparison with metazoan and yeast (DeGrasse *et al*., 2009). Further, an exhaustive interactome assigned *T. brucei* nucleoporins to discrete NPC substructures, which despite retaining similar protein composition also presented remarkable architectural differences (Obado *et al*., 2016; illustrated in Fig. 2). Briefly, while most elements of the inner core are conserved, multiple peripheral structures are highly dissimilar, possibly to accommodate divergent nuclear and cytoplasmic functions (Obado *et al*., 2016). *Tb*NPC is highly symmetric, with asymmetry only provided by its two nuclear basket Nups (Obado *et al*., 2016). Further, orthologues of cytoplasmic Nups or mRNA remodelling factors are absent in trypanosomes. Notably, *Tb*Nup76, likely the cytoplasm-specific Nup82/88 orthologue, localizes to both faces of the NPC (Obado *et al*., 2016). Overall, trypanosomes present substantial variation in the pore membrane proteins and the absence of critical components involved in mRNA export in fungi and animals. Additionally, there is evidence supporting a Ran-dependent system for mRNA export in trypanosomes, which suggests distinct mechanisms of protein and mRNA transport (Obado *et al*., 2016).

*Tb*Nup110 and *Tb*Nup92, the two components of the nuclear basket, are predicted to have predominantly coiled-coil structure and are likely to represent the Mlp/Tpr proteins of trypanosomes (Holden *et al*., 2014). Despite performing similar roles in chromosome segregation, *Tb*Nup92 has a restricted taxonomic distribution and appears to have a distinct evolutionary origin than Mlp. Further, unlike Mlp, there was no evidence for a role on the creation of transcriptional boundaries, consistent with trypanosome genome organization and gene expression control (Holden *et al*., 2014). However, *Tb*Nup92-knockout cells differentially expressed genes associated with RNA turnover, raising the interesting possibility that *Tb*Nup92 might associate with a particular subset of RNA-binding proteins (Holden *et al*., 2014).

Notably, in *T. brucei* as well as related organisms, a comprehensive analysis on whether there are changes in the NPC composition or structure following differentiation into different developmental stages is yet to be performed (Rout *et al*., 2017); and if such changes occur, whether those play a role in gene expression modulation is yet to be investigated.

#### Nuclear lamina

In mammals, NL is a meshwork consisting of A- and B-type lamins and lamin-associated proteins, which lines the inner nuclear membrane. In differentiated cells, lamin expression is critical to sustain nuclear architecture, prevent abnormal blebbing of the nuclear envelope, and position the NPCs (Dechat *et al*., 2008). NL can influence transcriptional activity and interact with a wide range of transcription factors; it is also involved in the compaction of peripheral chromatin (Shevelyov and Ulianov, 2019). Eukaryotic heterochromatin, which is mainly located at the nuclear periphery, is subdivided into densely packed constitutive heterochromatin, including pericentromeric and telomeric chromosomal regions, and the less condensed or so-called facultative heterochromatin located in chromosomal arms (Finn and Misteli, 2019). Chromosomal regions interacting with the NL are designated lamina-associated domains (LADs) have been identified in a wide-range of eukaryotes, from nematodes to humans, and contain mostly silent or weakly expressed genes (Shevelyov and Ulianov, 2019). This supports the idea that NL is a repressive nuclear compartment.

Lamin genes were found in metazoa but appeared to be absent in plants and unicellular organisms. In mammals, two major A-type lamins (lamin A and C) and two major B-type lamins (lamin B1 and B2) have been identified and characterized (Dechat *et al*., 2008). They are composed of a long central *α*-helical rod domain, flanked by globular N-terminal (head) and C-terminal (tail) domains, which self-assemble into higher-order structures whose basic subunit is a coiled-coil dimer (Dechat *et al*., 2008). Notably, aberrant lamin protein structure or expression can lead to irregular nuclei and abnormal gene expression. Indeed, hundreds of mutations have been identified in human lamins and linked to diseases, collectively known as laminopathies that include progeria and muscular dystrophies (Dechat *et al*., 2008). Interestingly, examples from yeast and plants suggest that alternative, non-lamin, molecular systems can construct an NL (Dechat *et al*., 2008).

In *T. brucei*, an analogous to vertebrate lamins, NUP-1 is a major component of the nucleoskeleton and plays a key role on heterochromatin organization at the nuclear periphery (DuBois *et al*., 2012; illustrated in Fig. 2). NUP-1 is a critical component of a stable network at the inner face of the trypanosome nuclear envelope, its depletion leads to abnormally shaped nuclei and disrupts NPCs and chromosomes organization (DuBois *et al*., 2012). NUP-1 affinity purification led to the identification of a second coiled-coil protein, designated NUP-2. Following NUP-2 depletion, NUP-1 is mislocalized and *vice versa*, strongly suggesting that NUP-1 and NUP-2 form a co-dependent network (Maishman *et al*., 2016). NUP-2 knockdown leads to severe fitness cost and a dramatic impact on nuclear architecture including severe changes to the nuclear envelope and chromosomal organization. Moreover, NUP-1 and NUP-2 are conserved across trypanosomes; from a structural and functional perspective, they behave similarly to lamins (Maishman *et al*., 2016).

**Fig. 3.**
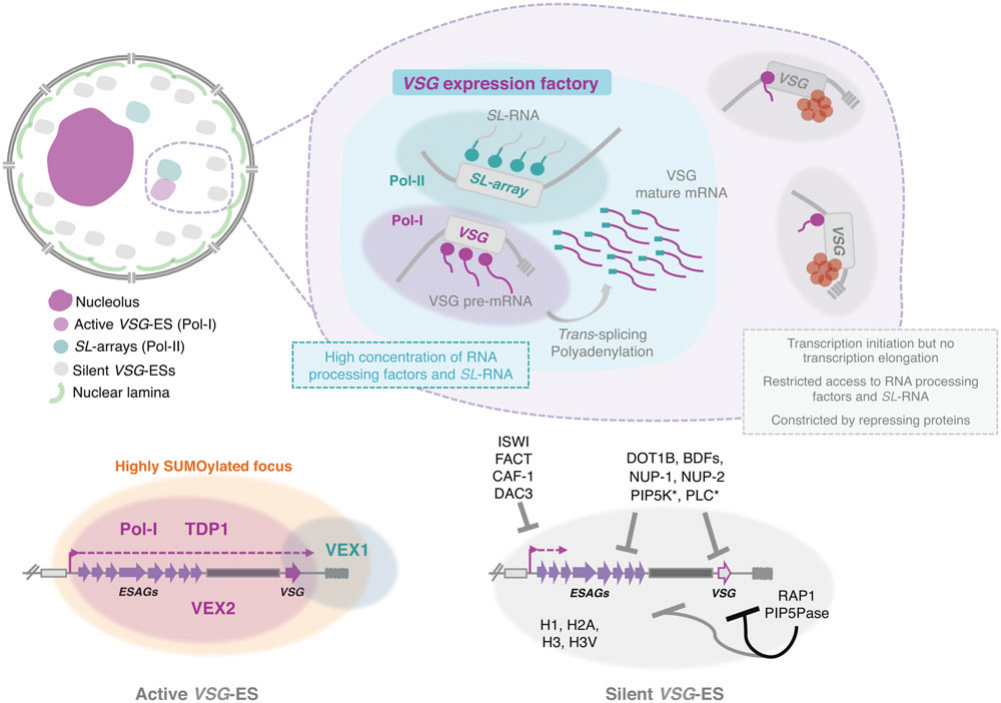
Nuclear organization and *VSG* expression in *T. brucei* bloodstream forms. The single active-*VSG* establishes a stable inter-chromosomal interaction with one of the *SL*-arrays. VEX2 orchestrates this spatial integration, which is critical to (1) sustain monogenic expression, (2) enhance RNA processing (Faria *et al*., 2020). The active-*VSG* gene is transcribed at very high levels by Pol-I generating the most abundant protein in the cell. Proximity to the *SL*-array likely leads to a high local concentration of *SL*-RNA therefore facilitating *trans*-splicing. It is possible that several factors associated with RNA processing (splicing, polyadenylation, etc.) are concentrated in this sub-nuclear compartment as well. The *SL*-array appears to function as a post-transcriptional enhancer and such control might extend beyond *VSG* genes (Faria *et al*., 2020). The active *VSG*-ES lies within a highly SUMOylated focus (López-Farfán *et al*., 2014); TDP1 is a high mobility group box protein that facilitates Pol-I transcription and is enriched at the active-ES (Narayanan and Rudenko, 2013). VEX2 and VEX1 form discrete protein condensates that associate with the active-*VSG* and the *SL*-array, respectively. The VEX complex, especially VEX2, sustains the exclusive interaction between a single *VSG*-ES and the *SL*-array; following its depletion, all *VSG*-ESs can access the *SL*-arrays and are derepressed (Faria *et al*., 2019, 2020). The silent *VSG-*ESs have more peripheral locations; transcription by Pol-I is initiated at the same rate as at the active-locus but transcription elongation is unsuccessful; these sites also have restricted access to RNA processing factors and substrates (Vanhamme *et al*., 2000; Kassem *et al*., 2014); several repressing factors associated with heterochromatin formation (red circles) sustain their inactive state. For instance, ISWI (Hughes *et al*., 2007), FACT (Denninger and Rudenko, 2014), CAF-1 (Alsford and Horn, 2012) or DAC3 (Wang *et al*., 2010) repress transcription near the promoter in silent ESs. Telomeric ES proteins, such as RAP1 (Yang *et al*., 2009) or PIP5Pase (Cestari *et al*., 2019), repress transcription of the whole ES, the repressive gradient is stronger near the telomeres (indicated by the darker line). Other repressive proteins include DOT1B (Figueiredo *et al*., 2008), bromodomain proteins (BDFs) (Schulz *et al*., 2015) and PIP5K and PLC, marked with an asterisk because they are the only ones that do not localize to the nucleus (Cestari and Stuart, 2015). Moreover, the integrity of the nuclear lamina is critical to maintain this repressive state (DuBois *et al*., 2012).

Notably, while the active *VSG*-ES resides within a transcription factory adjacent to the nucleolus (Navarro and Gull, 2001) the silent *VSG*-ESs are located at the extra-nucleolar nucleoplasm but at more peripheral locations in BSFs (Chavez *et al*., 1998; Landeira and Navarro, 2007; Fig. 2B and Fig. 3). Further, all *VSG*-ESs localize to the nuclear envelope and appear to form constitutive heterochromatin in insect-stage cells (Landeira and Navarro, 2007). This is consistent with the idea that the NL is a repressive compartment. Curiously, in *Plasmodium*, all silenced *var* genes localize in a series of clusters at the nuclear periphery, however, the transcription of the active *var* gene also occurs at a specific site at the nuclear periphery, where the activated gene moves away from the silenced clusters (Duraisingh *et al*., 2005; Freitas-Junior *et al*., 2005; Lemieux *et al*., 2013).

In *T. brucei*, NUP-1 plays a role on epigenetic control of developmentally regulated loci. Indeed, following NUP-1 knockdown, megabase chromosome telomeres reposition, multiple *VSG*-ESs become active, and the frequency of *VSG* switching increases (DuBois *et al*., 2012; Rout *et al*., 2017). Additionally, the active *VSG*-ES promoter fails to migrate to the nuclear periphery upon differentiation, and metacyclic *VSG*s are derepressed in insect-stage cells, both likely associated with the defective formation and/or maintenance of a repressive heterochromatin compartment (DuBois *et al*., 2012). Heterochromatin-based silencing in trypanosomes involves several proteins, such as ISWI, RAP1 and histone deacetylase (DAC) 3 (Hughes *et al*., 2007; Yang *et al*., 2009; Wang *et al*., 2010), whilst histone H1 participates in maintaining condensed chromatin in silenced regions (Povelones *et al*., 2012). Strikingly, *T. brucei* lacks H3K9me3, a well-characterized marker for heterochromatin, and heterochromatin-protein 1 (HP1) (Berriman *et al*., 2005). It is noteworthy that the misregulation of *VS*G and procyclin genes is quite modest following NUP-1 depletion (up to 10-fold) (DuBois *et al*., 2012); however, it demonstrates that NUP-1 and the trypanosome NL integrate a series of possibly multiple mechanisms that constrain the inactive *VSG*-ESs and reinforce their silent state.

### Membraneless nuclear bodies

Eukaryotic cells contain membraneless organelles, designated cellular bodies, which compartmentalize essential biochemical reactions and cellular functions. These bodies are generated by phase separation mediated by cooperative interactions between multivalent molecules (Strom and Brangwynne, 2019; Razin and Gavrilov, 2020). Well-characterized examples of such organelles in the nucleus are nucleoli, which are sites of rRNA biogenesis; Cajal bodies (CB), which are assembly sites for small nuclear ribonucleoproteins (RNPs); and nuclear speckles (NSs), which are storage compartments for RNA processing factors (Strom and Brangwynne, 2019; Razin and Gavrilov, 2020). Besides their ability to move throughout the nucleus, another fascinating feature of several nuclear bodies is their ability to form within the nuclear milieu without apparent support structures, again consistently with a self-organization model. Moreover, these organelles exhibit properties similar to liquid droplets, being able to undergo fission and fusion. In fact, mixtures of specific RNA and certain RNA-binding proteins are able to form phase-separated bodies *in vitro* (Guo *et al*., 2019; Hondele *et al*., 2019).

#### Nucleolus

The nucleolus is likely to be the most distinctive nuclear compartment, certainly the largest and the site of ribosome biogenesis where the 45S ribosomal repeats are clustered. Indeed, pre-rRNA transcription and processing as well as the assembly of the 40S and 60S complexes take place in this nuclear body (Hernandez-Verdun *et al*., 2010). In animals and plants, the nucleolus presents a tripartite substructure, which can be observed by electron microscopy. This tripartite substructure includes fibrillar centres (FC) surrounded by a dense fibrillar component (DFC); both embedded in the granular component, the biggest nucleolar subdomain composed of RNP granules. FC stores inactive rRNA genes, whereas DFC is electron dense given the high concentration of RNPs and is involved in early rRNA processing (Hernandez-Verdun *et al*., 2010).

Eukaryotic ribosomes are composed of 18S, 5.8S, 28S and 5S rRNA subunits and approximately 80 associated proteins. The four rRNA molecules are the main structural and catalytic components of the ribosome. In most eukaryotes, genes encoding for 18S, 5.8S, 28S are organized in tandem repeats, which are transcribed by RNA Polymerase I (Pol-I) into a primary transcript further processed into the mature 18S, 5.8S, 28S rRNAs (Hernandez-Verdun *et al*., 2010). Transcription occurs in the boundary between FC and DFC. 5S rRNA genes, on the other hand, are transcribed in the nucleoplasm by Pol-III (Hernandez-Verdun *et al*., 2010).

Similarly to other eukaryotes, the nucleolus is the most distinctive membraneless sub-nuclear body in trypanosomes and *Leishmania* parasites that can be easily observed by light and electron microscopy (Ogbadoyi *et al*., 2000; Nepomuceno-Mejía *et al*., 2010). Presently, FCs have not been identified in the nucleolus of these organisms, which presents a bipartite structure, similarly to other protozoa, yeast, invertebrates, fish and amphibians (Ogbadoyi *et al*., 2000; Nepomuceno-Mejía *et al*., 2010; illustrated in Fig. 2). Unlike more complex eukaryotes, during cell division in *T. brucei*, the nuclear envelope is preserved, chromatin does not condense and the nucleolus does not disassemble. As mitosis progresses, the nucleolus stretches, is pulled *via* the spindle fibres to opposite poles of the nucleus and ultimately divided into two independent structures (Ogbadoyi *et al*., 2000). This process occurs in the absence of intermediate structures such as prenucleolar bodies, found in other organisms (Hernandez-Verdun *et al*., 2010).

In *T. brucei* and similarly to other organisms, the biogenesis of ribosome subunits starts in the nucleolus and ends in the cytoplasm. The 5S rRNA is imported to the nucleolus very early in the biogenesis process and incorporated into the 90S pre-ribosome as an RNP complex; it later undergoes spatial rearrangement to facilitate subsequent maturation steps of the 60S subunit (Prohaska and Williams, 2009; Liu *et al*., 2016). The pre-60S particle is translocated from the nucleus to the cytoplasm through interactions between P34 and P37and exportin 1 and Nmd3, as well as r-proteins uL3 and uL11 (Prohaska and Williams, 2009). The biogenesis of the 40S subunit in *T. brucei* occurs very similar to what has been described in yeast (Ferreira-Cerca *et al*., 2007). Interestingly, this subunit contains a trypanosomatid-specific helical structure that has been proposed to participate in translation initiation by interacting with the *SL*-sequence and its unusually modified cap (Hashem *et al*., 2013).

In humans, the nucleolus has been associated with multiple functions that extend beyond ribosome biogenesis, one being a cellular stress sensor (Rubbi and Milner, 2003). Studies in trypanosomes suggest this may be the case in trypanosomatid parasites as well (Elias *et al*., 2001; Barquilla *et al*., 2008). Moreover, the nucleolus appears as a largely self-organized structure. Indeed, its integrity relies on both active Pol-I transcription and high interactivity between ribosomal components (Raska *et al*., 2006). Interestingly, ectopic expression of rRNA leads to the formation of micronucleoli in *Drosophila* (Karpen *et al*., 1988), again consistently with a model of self-organized nuclear compartmentalization. In trypanosomes, specifically, depletion of Pol-I-specific subunits leads to abnormal nucleoli (Devaux *et al*., 2007) and depletion of TOR1 kinase leads to Pol-I and nucleolar dispersion, most likely as a consequence of Pol-I transcription inhibition (Barquilla *et al*., 2008). In *T. cruzi*, development from a proliferative to non-proliferative stage, which is associated with a pronounced drop in transcriptional activity, is also accompanied by nucleolar dispersion (Elias *et al*., 2001).

Further details on nucleolar structure and function in trypanosomatid parasites have been recently reviewed by (Martínez-Calvillo *et al*., 2019).

#### Nuclear speckles and Cajal bodies

In complex eukaryotes such as animals and plants, CBs are involved in the post-transcriptional maturation of small nuclear (snRNAs) and small nucleolar RNAs (snoRNAs) and the biogenesis of nuclear RNPs, including some nucleolar proteins, snoRNPs and snRNPs (Sawyer *et al*., 2016). The number of CBs varies across cell types and at a single-cell level within the same cell type (in mammalian cells typically 0–10 CBs per nucleus, ranging 0.1–2 *μ*m in diameter). CBs are more abundant in cells with high transcriptional activity and are highly dynamic but structurally stable structures. They continuously exchange components into and out of the domain in response to changes in the cellular environmental (Sawyer *et al*., 2016). Interestingly, components of the SNAPc complex were reported to be enriched within the CB, suggesting a strong link between snRNA gene transcription and CBs. Several studies also indicate that CBs influence the levels and processivity of factors crucial for efficient RNA splicing; indeed CBs may influence splicing kinetics through different pathways (Sawyer *et al*., 2016).

Coilin and the nucleolar protein Nopp140 are the two key markers of CBs. Coilin has been implicated in the link between the nucleolus and CBs; indeed CBs are frequently detected at the nucleolar periphery and even within nucleoli. Coilin is a key structural component of CBs, is involved in RNP metabolism within these nuclear bodies and it also appears to have a role on general chromatin organization (Machyna *et al*., 2015). Its N-terminal domain is responsible for the self-oligomerization activity, truncation or mutation of phosphorylation sites in the conserved C-terminal region leads to a dramatic alteration in the number of CBs (Shpargel *et al*., 2003). On the other hand, Nopp140 does not localize strictly to CBs and it appears to serve generally as a chaperone for RNPs; it moves between the nucleolus and the CBs, but also between the nucleolus and the cytoplasm (Isaac *et al*., 1998). Indeed, it not only interacts with coilin, but also associates with several nucleolar proteins (Isaac *et al*., 1998).

*Trypanosoma brucei* appears to lack a coilin homologue and TbNopp140 is strictly nucleolar, strongly suggesting that CBs, in the strict sense of the definition, are absent in these parasites (Berriman *et al*., 2005; Kelly *et al*., 2006) (Fig. 2). Additionally, *T. brucei* possesses two homologues of Nopp140, a canonical Nopp140 and a Nopp140-like protein, both are phosphorylated and co-immunoprecipitate with Pol-I and might play a role on nucleoplasmic snoRNPs shuttling (Kelly *et al*., 2006). Given the absence of CBs, it has been proposed that in *T. brucei* RNPs are probably assembled in analogous bodies: a possible candidate was a compartment identified as Spliced-leader-associated RNA (SLA1)-containing subnuclear site that did not colocalize with *SL*-RNA (Hury *et al*., 2009). SLA1 guides the pseudouridylation at position −12 (relative to the 5′ splice site) of the *SL*-RNA in all trypanosomatid species.

NSs or splicing speckles were originally discovered as sites for splicing factor storage and modification and were later revealed to play a general role in RNA metabolism. Subsequently, numerous proteins involved in epigenetic regulation, chromatin organization, DNA repair and RNA modifications were found in NSs (Galganski *et al*., 2017). Similar to other membraneless bodies with liquid-like properties, NSs are characterized by the dynamic exchange of components within the nucleoplasm, sharing some proteins with other nuclear bodies (Galganski *et al*., 2017).

In trypanosomes, *trans*-splicing occurs for every single mRNA, there are only two known *cis*-spliced introns in *T. brucei*; both mechanisms seem to require the spliceosome. Notably, trypanosomes encode for all snRNA and many spliceosomal proteins described in other eukaryotes but also encode for a few specific factors (Palfi *et al*., 2000; Ambrósio *et al*., 2009; Preusser *et al*., 2009; also reviewed by Günzl, 2010; Michaeli, 2011 and Clayton, 2019). Interestingly, splicing factors such as Prp31, SmE, SSm2-1, PRP19 and SPF27 have a speckle-like organization and appear to be compartmentalized in specific nuclear areas (Liang *et al*., 2006; Tkacz *et al*., 2007; 2010; Ambrósio *et al*., 2015; illustrated in Fig. 2B).

Recent advances in more complex eukaryotes suggested that NSs facilitate integrated regulation of gene expression (Galganski *et al*., 2017). A substantial fraction of the mammalian genome is preferentially organized around nuclear bodies such as the nucleolus and NSs; these bodies have been proposed to act as inter-chromosomal hubs that shape the overall packaging of DNA in the nucleus (Quinodoz *et al*., 2018). Additionally, many active genes reproducibly position near NSs, but the nature of such associations had remained unclear until recently, when a study linked them to stochastic gene expression amplification (Kim *et al*., 2020). Whether similar associations are present and play a role in genome organization and gene expression in trypanosomes and related organisms remains to be explored.

In summary, compartmentalization within the nucleoplasm enables functional specialization; in fact, key nuclear functions such as transcription or RNA processing are not homogeneously distributed throughout the nucleus. In the next chapter, I will specifically cover the current knowledge on transcription regulation and compartmentalization in trypanosomes.

## Transcription regulation

To our knowledge, all trypanosomatids employ primarily polycistronic transcription, where multiple open reading frames with no functional association are transcribed in tandem. Evidence suggests that the position within the PTU is associated with messenger RNA (mRNA) copy number (Kelly *et al*., 2012). The nascent RNAs are processed into mature mRNAs, through a combination of *trans*-splicing and polyadenylation (reviewed by Günzl, 2010; Michaeli, 2011; Clayton, 2019). Notably, mature mRNAs bear an unusual hypermethylated 5′ cap structure (Bangs *et al*., 1992). The genome is therefore constitutively transcribed and mRNA abundance is primarily controlled at the post-transcriptional level in striking contrast with more complex eukaryotes, where a specific promoter usually regulates the transcription of each gene (Koumandou *et al*., 2008).

Exceptions to this mechanism are the genomic loci encoding for highly abundant surface-exposed antigens, *VSG*s and procyclins: these loci are transcribed at very high levels by Pol-I and not Pol-II, which transcribes the majority of PTUs. Both *VSG*s and procyclins expression is developmentally regulated, the former expressed in the mammalian-stage and the latter in the insect-stage (Navarro *et al*., 2007; Daniels *et al*., 2010).

### RNA polymerase I (Pol-I)

*Trypanosoma brucei* is the only organism known to have evolved a multifunctional Pol-I system that is used for rRNA synthesis and for the expression of highly abundant antigens (Günzl *et al*., 2003). As previously mentioned, *VSG*s and procyclins are strongly developmentally regulated and therefore Pol-I transcription in *T. brucei* is intimately linked to differentiation between different life cycle stages as well as antigenic variation in the mammalian host, which is critical to sustain persistent infections.

In the vast majority of eukaryotes, Pol-I is recruited to simple promoters, which contain an upstream element located 100 bp from the transcription start site. Such promoters are exclusively used for rRNA gene expression, specifically the 45S rRNA precursor, further processed into 18S, 5.8S and 28S rRNA. Two protein complexes, the selectivity factor 1 (SL1) and the upstream binding factor (UBF), are essential for Pol-I recruitment to the rRNA promoter (Russell and Zomerdijk, ). The interaction between Pol-I and SL1 is mediated by a single polypeptide named RRN3 in humans; a UBF dimer is further required to activate rRNA transcription. In yeast, RRN3 is conserved, whereas the three subunits of the core factor (the functional equivalent of SL1) and the six UBF subunits share no sequence similarity with the mammalian counterparts (Russell and Zomerdijk, ). Recent CryoEM studies suggest that, unlike the Pol-II system, promoter specificity relies on a distinct ‘bendability’ and ‘meltability’ of the promoter sequence that enables contacts between initiation factors, DNA and polymerase (Engel *et al*., 2017). In eukaryotic cells, although the number of rRNA genes is much lower than the number of protein-coding genes, Pol-I transcription usually accounts for more than 50% of the total transcriptional activity, which results from impressively high transcription initiation rates. Notably, mammalian Pol-I is unable to synthesize functional mRNA (Russell and Zomerdijk, ).

Similarly to all eukaryotes, *T. brucei* Pol-I transcribes the 45S rRNA precursor in the nucleolus; however, it also transcribes procyclins and *VSG*s mRNAs from perinucleolar and extra-nucleolar locations, respectively (Navarro and Gull, 2001) (Figs 2B and 3). The rRNA, *VSG* and procyclin gene promoters are structurally different, suggesting that they recruit different transcription factors. Since the last two promoters are absent in related organisms *T. cruzi* and *Leishmania* spp., one would expect to find *T. brucei*-specific proteins for *VSG* and procyclin gene transcription.

In *T. brucei*, both bioinformatics and biochemical analyses have unravelled 10 out of 12 Pol-I subunits: RPA1, RPA2, RPC40, RPB5z, RPB6z, RPB8, RPC19, RPB10z and RPA12 (Walgraffe *et al*., 2005; Nguyen *et al*., 2006). RPB5z, RPB6z and RPB10z are RPB5, RPB6 and RPB10 paralogs, respectively. Further, *T. brucei* presents functional diversification of isoforms that are conventionally shared RNA polymerase subunits (Devaux *et al*., 2007). *Trypanosoma brucei* also has a specific component (or a divergent orthologue of yeast RPA43), RPA31, which is critical for Pol-I transcription and cell viability (Walgraffe *et al*., 2005; Nguyen *et al*., 2007). Although conceivable, there is no evidence that RPA31, RPB5z, RPB6z and RPB10z play a specific role on mRNA transcription by Pol-I in *T. brucei*.

The class I transcription factor A (CITFA) has been identified in *T. brucei*; its purification led to the identification of seven novel subunits, termed CITFA-1 to -7, plus the dynein light chain DYNLL1 (also known as LC8) (Brandenburg *et al*., 2007; Nguyen *et al*., 2012). CITFA binds rRNA, *VSG* and procyclin promoters and therefore is a general Pol-I transcription factor in *T. brucei*; its depletion is unsurprisingly lethal (Brandenburg *et al*., 2007). Further, TDP1, a high motility group box containing protein, which facilitates Pol-I transcription, is highly enriched at the active *VSG*-ES (compared to silent) and in the nucleolus; a blockade in TDP1 synthesis results in a pronounced reduction of Pol-I-derived transcripts (Narayanan and Rudenko, 2013). TDP1 overexpression was sufficient to open the chromatin of silent *VSG*-ESs and disrupt *VSG* monogenic expression (Aresta-Branco *et al*., 2019). Moreover, ELP3B was identified as a specific negative regulator of rRNA transcription (no impact on *VSG* transcription); these observations extend the roles of the Elp3-related proteins to Pol-I transcription units, as they are usually associated with Pol-II transcription in humans and yeast (Alsford and Horn, 2011).

Notably, all proteins involved in *T. brucei* Pol-I transcription identified so far are conserved among all trypanosomatids, suggesting that they fulfil general Pol-I functions (Walgraffe *et al*., 2005; Nguyen *et al*., 2006; Devaux *et al*., 2007). However, it is entirely possible that these common factors evolved specific functions for protein-coding gene transcription in *T. brucei*. Nevertheless, how *T. brucei* Pol-I acquired the ability to transcribe mRNA remains mysterious.

### RNA polymerase II (Pol-II)

Pol-II synthesizes pre-mRNAs and U-rich short nuclear RNAs (snRNAs). The latter form the core of the spliceosome, involved in processing pre-mRNAs into mature mRNAs. In both cases, the 5′ ends are capped, which requires adding m^7^G to the 5′ triphosphate end of the primary transcript and takes place co-transcriptionally (Proudfoot *et al*., 2002). There are several other co-transcriptional activities, which are assigned to specific subunits or domains within these subunits (Proudfoot *et al*., 2002). *Trypanosoma brucei* Pol-II produces both pre-mRNAs and the spliced leader (*SL*)-RNA, the latter is detrimental for *trans*-splicing.

Eukaryotic Pol-II enzymes usually contain 12 subunits, designated RPB1 to RPB12. Specifically, RPB1, RPB2, RPB3 and RPB11 are considered the functional and structural core subunits. Additionally, RPB4 to RPB10 and RPB12 usually contribute to Pol-II ability to respond to activators and tightly bind promoter regions (Proudfoot *et al*., 2002). The 12 Pol-II subunits could be identified in *T. brucei*; RPB1, RPB2, RPB3 and RPB11 were also considered the functional and structural core (Das *et al*., 2006; Devaux *et al*., 2006). Interestingly, trypanosomes have two isoforms of RPB5 and RPB6 (Das *et al*., 2006; Devaux *et al*., 2006). RPB1 is the largest subunit in the *T. brucei* enzyme and also the most fascinating (Evers *et al*., 1989). One of the most remarkable characteristics is the non-structured carboxyl end of the polypeptide, which deviates from the heptapeptide repeat of YSPTSPS of varying length that is characteristic of yeast and mammalian proteins. This repeat is generally involved in the modulation of multiple co-transcriptional processes that include capping, splicing, elongation, polyadenylation and nuclear export, through coordinated kinetic alterations in the phosphorylation of its serines and threonines (Proudfoot *et al*., 2002). Despite being non-repetitive, the trypanosome carboxyterminal is phosphorylated and essential for transcription (Evers *et al*., 1989). *Trypanosoma brucei* cdc2-related kinase 9 (CRK9) was found to be responsible for RPB1 phosphorylation, however, surprisingly, when silencing CRK9, there was no impact on Pol-II transcription or co-transcriptional m^7^G capping. Instead it led to a block of *trans*-splicing caused by hypomethylation of the *SL*-RNA unique cap4 (Badjatia *et al*., 2013).

In many organisms, a crucial regulatory point of gene expression is transcription initiation, which requires the formation of a pre-initiation complex that includes multiple proteins that interact with Pol-II. Such transcription factors include TFIIA, TFIIB, TFIID, TFIIE, TFIIF and TFIIH, which recruit and position Pol-II at promoter sequences (Hahn, 2004). The only canonical Pol-II promoter in *T. brucei* is the *SL*-RNA promoter. In this organism, the identification of general transcription factors was challenged by their extremely divergent amino acid sequences from those of their eukaryotic counterparts. The first transcription factor purified and characterized was a trimeric SNAPc that formed a larger complex with TATA-binding protein, the small subunit of TFIIA (TFIIA2), and a sixth protein (TFIIA1) (Das *et al*., 2005; Schimanski *et al*., 2005). This was followed by the identification of TFIIB, TFIIH, TFIIE; later, a TFIIH-associated complex of nine subunits was discovered, and despite exhibiting no motif or sequence conservation that could reveal its identity, it structurally resembled the head module of the much larger mediator complex of other eukaryotes (Schimanski *et al*., 2006; Lee *et al*., 2007, 2010). More recently, a TFIIF-like or TFL complex has been identified, strongly indicating that trypanosomatids possess a full set of RNA Pol-II general transcription factors, only very divergent from their mammalian and yeast counterparts (Srivastava *et al*., 2018). All these factors are required for *SL*-RNA transcription and trypanosome viability, but their role, if any, on the transcription of protein-coding genes remains unknown.

In *T. brucei*, ubiquitously expressed genes lack well-defined Pol-II promoter motifs, with the exception of the spliced-leader RNA promoter. Indeed, the so-called Pol-II *disperse* promoters lack conserved sequence motifs and tight regulation; however, they are defined by specific chromatin structures. In *T. brucei* for instance, GT-rich promoters were recently proposed to drive transcription and promote the targeted deposition of the histone variant H2A.Z, showing that even highly dispersed, unregulated promoters might contain specific DNA elements that are able to induce transcription (Wedel *et al*., 2017).

Additionally, Pol-II transcription termination is a tightly regulated process and critical to prevent the elongating Pol-II complex from interfering with the transcription of downstream genes. In kinetoplastid flagellates, the modified base *β*-D-glycosyl-hydroxymethyluracil (J) replaces a small percentage of thymine residues, mostly in telomeric regions and is synthesized at the DNA level *via* the precursor 5-hydroxymethyluracil. In *T. brucei* for instance, base J is exclusively present in the BSF. Notably, in *T. brucei* and *Leishmania major*, base J and H3.V are enriched at sites involved in Pol-II termination. Loss of base J and H3.V led to transcription read-through (Reynolds *et al*., 2016; Schulz *et al*., 2016). Recently, a novel base J-binding protein complex involved in Pol-II transcription termination has been identified (Kieft *et al.*, 2020).

Overall, trypanosomes appear to have limited control over Pol-II transcription initiation, and therefore most of the gene expression control is thought to be post-transcriptional.

### RNA polymerase III (Pol-III)

Pol-III is responsible for the transcription of a number of small non-coding RNAs that play a role in translation (tRNA and 5S rRNAs) and other cellular processes (7SL RNA). In *T. brucei*, tRNA genes can be found widely spread throughout large directional gene clusters on megabase chromosomes, 5S rRNA genes are clustered in chromosome 8 (Berriman *et al*., 2005).

### Expression factories

#### The *SL*-RNA expression factory

Given that *SL*-RNA must be added to the 5′ end of every single mRNA in *T. brucei*, *trans*-splicing relies on large quantities of *SL* transcripts generated by Pol-II transcription from a diploid tandem-repeat locus. Indeed, Pol-II largest subunit is highly concentrated at the *SL*-RNA genomic loci (illustrated in Fig. 2B). In *T. cruzi* and *Leishmania tarentolae*, a single focus is observed possibly due to pairing of both alleles (Dossin and Schenkman, 2005). In contrast, in *T. brucei*, two distinct foci could be detected in G1 cells indicating that the two *SL*-arrays occupy distinct chromosome territories (Uzureau *et al*., 2008). In *T. cruzi*, the Pol-II focus disperses following treatment with transcription inhibitors (Dossin and Schenkman, 2005), suggesting that the high concentration and organization of Pol-II around the *SL*-arrays depends on active-transcription and therefore is not a predefined nuclear structure.

Moreover, *SL*-RNA transcripts concentrate in a nuclear area that colocalizes with the snRNP protein SmE and SLA1 RNA, an RNA involved in the *SL*-RNA modification. This strongly suggests that there is a spatially defined *SL*-RNP factory in the nucleoplasm (Tkacz *et al*., 2007). When labelling active transcription through BrdU incorporation, a broader distribution of extra-nucleolar transcriptional activity can be observed apart from the *SL*-RNA arrays (although that accounts for Pol-III as well) (Daniels *et al*., 2010; illustrated in Fig. 2B). One would expect that capping enzymes and cap methyltransferases would concentrate at *SL*-RNP factories, which is difficult to extrapolate from the localization data currently available and will therefore require a more detailed analysis, possibly with higher resolution microscopy.

#### The *VSG* expression factory

African trypanosomes and their VSGs are a fine example of extreme biology and have led to several groundbreaking discoveries, such as *trans*-splicing, mRNA transcription by Pol-I or GPI anchors (Navarro *et al*., 2007; Duraisingh and Horn, 2016). Notably, recent studies on *VSG* expression in *T. brucei* have revealed interesting features regarding genome architecture and nuclear compartmentalization that hint to unknown layers of gene expression control in these organisms.

The single active *VSG* gene generates the most abundant protein in the cell (approximately 10% of the total proteome), which results from a combination of high levels of transcription by Pol-I and multiple mechanisms of post-transcriptional control (Navarro and Gull, 2001; Günzl *et al*., 2003; do Nascimento *et al*., 2020; Viegas *et al*., 2020). This renders trypanosomes and their *VSG*s an amenable model system to study mechanisms underpinning single gene choice, which are not fully understood in any eukaryote. Indeed, monogenic expression is one of the greatest outstanding mysteries of eukaryotic gene expression. For instance, it also underpins singular expression of antigen and olfactory receptors, responsible for the specificity of the immune response and the sense of smell in mammals, respectively (Monahan and Lomvardas, 2015; Outters *et al*., 2015).

Interestingly, it was unclear whether genome architecture and specifically genome position played a role in gene expression control in trypanosomes and related organisms. However, *T. brucei* somehow employs a mechanism of monogenic antigen transcription in the absence of controlled transcription initiation and canonical enhancer sequences. Indeed, Pol-I transcription is initiated at the same rate at all *VSG*-ESs, however transcription elongation is restricted to the active-*VSG*-ES (Vanhamme *et al*., 2000; Kassem *et al*., 2014). Additionally, RNA maturation seems to be somehow restricted to the active *VSG*-ES suggesting that access to RNA processing factors or substrates might be limiting (Vanhamme *et al*., 2000; Kassem *et al*., 2014).

Notably, while the silent *VSG*-ESs were located at more peripheral locations (Chaves *et al*., 1998; Landeira and Navarro, 2007), the active *VSG*-ES was included within an extra-nucleolar structure (although in close proximity to the nucleolus), designated the expression-site body (ESB), a transcription factory that contains a local reservoir of Pol-I (Navarro and Gull, 2001) (Fig. 3). This exclusion from the nucleolus is independent of the promoter, as swapping the *VSG* promoter by an rRNA promoter did not lead to nucleolar incorporation (Chaves *et al*., 1998), suggesting that other DNA elements/factors are required for targeting.

The ESB emerged as the defining structure that sustained *VSG* monogenic expression, accommodating a single *VSG*-ES at a time. In fact, if two *VSG*s were simultaneously active, a dynamic colocalization with the ESB was observed (Chaves *et al*., 1999; Budzak *et al*., 2019). However, the mechanisms for targeting the active-*VSG* ES to the ESB as well as the protein composition and the exact DNA sequences incorporated within this structure have remained elusive. Although the complete molecular understanding is yet to be achieved, several major advances have taken place in the recent years.

Notably, the single active-*VSG* displays a specific inter-chromosomal interaction with a major mRNA splicing locus, one of the *SL*-RNA arrays, and this specific nuclear arrangement is critical to sustain *VSG* monogenic expression (Faria *et al*., 2020). Specifically, the single active-*VSG* is expressed within a dedicated sub-nuclear compartment harbouring the Pol-I transcribed antigen-coding gene and the Pol-II transcribed *SL*-array and their respective associated factors to ensure (1) monogenic antigen transcription and (2) efficient mRNA splicing (Faria *et al*., 2020) (Fig. 3). The *VSG* exclusion proteins 1 and 2 (VEX1 and VEX2), which form discrete protein condensates in the nucleus in BSFs specifically, associate with the *SL*-RNA array and the active-*VSG* ES, respectively (Faria *et al*., 2020) (Fig. 3). VEX1 was identified through a genetic screening (Glover *et al*., 2016) and VEX2 through VEX1 affinity purification (Faria *et al*., 2019). From the two proteins, VEX2, an RNA-helicase, has the most critical role on *VSG* monogenic expression: following its depletion, the ESB collapses and trypanosomes simultaneously transcribe all *VSG*-ESs, subsequently exposing multiple VSGs on their surface (Faria *et al*., 2019). Further, following VEX2 knockdown, all *VSG*-ESs can access the *SL*-RNA arrays, showing that VEX2 somehow sustains this dedicated sub-nuclear compartment and an exclusive association between the single active-*VSG* and the *SL*-array (Faria *et al*., 2020). Additionally, besides maintaining an exclusive interaction between the active-*VSG* and the *SL*-array, VEX2 appears to fine-tune gene expression at the active-*VSG* locus (Faria *et al*., 2019, 2020). It is tempting to speculate that it orchestrates a specific chromatin configuration that maximizes the interaction between the *VSG* gene itself (not the promoter or the ES-associated genes) and the *SL*-array.

### Phase separation and transcriptional control

More recently, liquid–liquid phase separation (LLPS) has been proposed (opinion piece by Hnisz *et al*., 2017) and later demonstrated (Guo *et al*., 2019) to be a major regulatory mechanism for enhancer-mediated transcriptional control in mammalian cells. Enhancers are short (50–1500 bp) DNA regulatory elements that activate the transcription of specific genes to a much higher level than would be the case in their absence; they function as a platform for the recruitment of activators, transcription factors and the RNA polymerase components. These DNA elements have a distal location and are brought in proximity to the target gene through chromatin loops. Notably, nucleation of phase-separated multi-molecular assemblies at enhancer sequences can explain the formation of super-enhancers (clusters of enhancers; sometimes hundreds), their high sensitivity to transcription inhibition, enhancer-mediated patterns of transcriptional bursts and simultaneous activation of multiple genes by the same enhancer (Hnisz *et al*., 2017). Notably, computational simulations have shown that LLPS can explain experimental observations that traditional models for transcriptional control cannot (Hnisz *et al*., 2017).

Enhancer sequences have never been found in trypanosomes and related parasites, and given their polycistronic transcription and overall lack of controlled transcription initiation, such mechanisms were thought to be unlikely to operate. But is this really the case? Indeed, it was unclear whether and how genome architecture and genome position played a role in gene expression in these parasites. Could it be that trypanosomes evolved unconventional enhancers? This will be addressed in the ‘Discussion’ section.

## Discussion

Despite the many open questions, previous studies following the depletion of several chromatin-associated factors (reviewed by Cestari and Stuart, 2018) and the recently unveiled association between the active-*VSG* and the *SL*-array unequivocally demonstrate that genome architecture does play a role in *VSG* monogenic transcription in *T. brucei*. Further, spatial proximity to RNA-processing centres might be a conserved mechanism for post-transcriptional enhancement of gene expression but this had not previously been linked to inter-chromosomal interactions.

It is possible that all *VSG*-ESs are able to stochastically interact with the *SL*-arrays and compete for a limited pool of VEX2, which will then stabilize an exclusive interaction between a single *VSG* locus and the *SL*-array. This would render VEX2 a limiting factor, which is supported by its low abundance and tight regulation (Faria *et al*., 2019). Interestingly, this could be explained by an LLPS model (Hnisz *et al*., 2017; Guo *et al*., 2019); indeed, phase-separating proteins were shown to be capable of generating stable sub-nuclear structures from dynamic interactions in mammals (Shin *et al*., 2018). Multiple studies have shown that high local concentrations of specific proteins and nucleic acids (where RNAs appear to be major players) and cooperative interactions among these molecules are implicated in the formation of phase-separated bodies (Shin *et al*., 2018; Guo *et al*., 2019). Recently, a family of RNA helicases has been identified as major regulators of the assembly of sub-nuclear compartments through LLPS (Hondele *et al*., 2019); therefore, it is tempting to speculate this might be the case of VEX2. In fact, specific post-translational modifications can trigger nucleation of phase-separated bodies; curiously, the active-ES resides within a hot spot of highly SUMOylated proteins (López-Farfán *et al*., 2014). Notably, the global role, if any, of LLPS and phase-separating proteins on genome organization in Trypanosomatids is yet to be investigated.

Inter-chromosomal interactions were thought to have a stochastic nature, indeed the existence of stable inter-chromosomal interactions has been a subject of debate as they were thought to be difficult to re-establish following cell division, possibly relying on error-prone mechanisms (Finn and Misteli, 2019). Consequently, their role on gene expression was rather dubious. The only other known stable interaction occurs in a terminally differentiated cell, and very interestingly, in another system subject to allelic exclusion. Indeed, olfactory neurons possess a multi-chromosomal super-enhancer that associates with the single active olfactory receptor gene (Monahan *et al*., 2019). In trypanosomes, the association of the active-*VSG* with the *SL*-array appears reminiscent, but classic transcriptional enhancement was replaced by what appears to be post-transcriptional enhancement instead. Despite the attractive theoretical reasons for the presence of such an enhancer in malaria-causing parasites, Hi-C analysis was unable to identify such an element in the *P. falciparum* genome (Lemieux *et al*., 2013).

In trypanosomes, proximity to the *SL*-array is likely to provide post-transcriptional enhancement due to a high local concentration of *SL*-RNA. A substantial amount of *SL*-RNA is therefore hijacked, so that RNA processing can keep pace with the high rate of transcription provided by Pol-I (Fig. 3). Notably, it will be interesting to identify other active *VSG*-ES-associated factors that take part in this antigen expression factory: it is entirely conceivable that a number of splicing factors and enzymes involved in polyadenylation might be concentrated in this compartment. This certainly adds a layer of post-transcriptional control that had not been previously characterized. Moreover, this association is also reminiscent of those between highly transcribed chromosome regions and NSs in mammals (Quinodoz *et al*., 2018; Kim *et al*., 2020). Whether the high transcription rate is the cause or a consequence of such association remains debatable. Similarly in *T. brucei*, whether the association with the *SL*-RNA array precedes the activation of the *VSG* locus, or whether it occurs afterwards *merely* providing post-transcriptional enhancement, remains unclear. Notably, in other organisms, co-transcriptional RNA processing can affect transcription elongation rates (Kornblihtt *et al*., 2004). How a specific *VSG* gene is activated over the other possible alleles remains a mystery, and those early events underpinning the establishment of an active transcriptional state are incredibly difficult to capture. In *Plasmodium*, for instance, antisense long-non-coding-RNAs play a key role in regulating *var* gene activation and mutually exclusive expression (Amit-Avraham *et al*., 2015).

Certainly several mechanisms simultaneously operate to constrain the inactive *VSG*-ESs and prevent their derepression. For instance, heterochromatin-based silencing in trypanosomes involves, among others, ISWI, RAP1 and histone deacetylase (DAC) 3 (Hughes *et al*., 2007; Yang *et al*., 2009; Wang *et al*., 2010; reviewed by Duraisingh and Horn, 2016; reviewed by Cestari and Stuart, 2018) (Fig. 3). The histone tri-methyltransferase DOT1B that targets H3K76, for instance, is required for rapid *VSG*-ES silencing and for an efficient transition from an active to a silent state (Figueiredo *et al*., 2008). Also, both the integrity of the NL and histone H1 are critical to maintain condensed chromatin in silenced regions (DuBois *et al*., 2012; Povelones *et al*., 2012). Strikingly, *T. brucei* lacks H3K9me3, a well-characterized marker for heterochromatin, and HP1 (Berriman *et al*., 2005), which plays a key role in *var* gene silencing in *Plasmodium* (Brancucci *et al*., 2014). Further, in *Plasmodium*, the histone methyltransferase SET10 colocalizes with the active *var* gene (Volz *et al*., 2012) and NAD(+)-dependent histone deacetylases, Sir2A and Sir2B, are required for silencing of different *var* gene subsets (Tonkin *et al*., 2009), but these histone modifiers do not appear to affect *VSG* silencing (Alsford *et al*., 2007). Indeed, in both trypanosomes and malaria-causing parasites, repressive heterochromatin plays a critical role in silencing all but one antigen-coding gene for successful antigenic variation. However, different chromatin remodellers, histone readers/erasers and histone chaperones appear to be involved in this process in trypanosomes and *Plasmodium* (reviewed by Duraisingh and Horn, 2016).

In a broader perspective, post-transcriptional enhancement of gene expression through spatial proximity to RNA-processing centres might be particularly relevant in less complex eukaryotes, where canonical transcriptional enhancers have not been identified, and particularly in Trypanosomatids, where transcriptional regulation is limited. Nonetheless, it remains to be investigated whether this type of regulation extends beyond *VSG*s in *T. brucei*, and whether it plays a broader role in gene expression control in kinetoplastids.

Notably, in *T. brucei*, Hi-C and ChIP-Seq analyses revealed that other highly transcribed loci (e.g. tandem arrays that encode for histones, tubulin, heat shock proteins, etc.) can interact with the *SL*-RNA array in the mammalian-infective stage (Faria *et al*., 2020). Moreover, in insect-stage *T. brucei*, procyclin coding loci also interact with the *SL*-RNA array (Faria *et al*., 2020). Given the fact that Hi-C is a very sensitive technique, it can capture strong and stable but also stochastic and transient interactions (Finn and Misteli, 2019); therefore, it will be interesting to investigate whether the interactions above are stochastic or whether they are associated with stable and heritable structures at the single-cell level. In other words, are there any other transcription/splicing factories in *T. brucei* and possibly in other related parasites? Could the *SL*-array act as an unconventional and post-transcriptional enhancer? The fact that the tubulin gene loci in *T. cruzi* do not colocalize with the *SL*-RNA arrays (Dossin and Schenkman, 2005) does not completely rule out this idea. This is not inconsistent with such interactions being transient and therefore more difficult to capture my microscopy, but could also mean that strong and stable interactions might be restricted to specific gene families and specific developmental stages, possibly depending on transcriptional activity. Additionally, it is very interesting that in *T. brucei*, the two *SL*-arrays occupy distinct chromosome territories, essentially there are two *SL*-RNA expression factories: is it because one is permanently used to sustain the expression of the active-*VSG* gene?

In mammals, a high degree of heterogeneity in genome organization has been observed, suggesting that individual cells in a population can assume many distinct, albeit related, spatial conformations (Finn *et al*., 2019). Notably, such variability does not mean that chromatin organization has no functional relevance, but rather suggests that structural heterogeneity may be another layer impacting gene expression (Finn and Misteli, 2019). In *Plasmodium* for instance, the 3D genome structure appears to be strongly connected with the transcriptional activity of specific gene families throughout the life cycle (Bunnik *et al*., 2018). Whether such variability can be observed in Trypanosomatid parasites and how that might modulate gene expression at the single-cell level and in different developmental stages remains to be unravelled.

## Future directions

*Trypanosoma brucei* genome sequencing was a phenomenal turning point that marked the beginning of a new era of research. Since then, sequencing technology, gene-editing tools, imaging and affinity purification techniques have massively evolved, allowing us to experimentally tackle long-standing questions that had been previously untrackable.

Similarly to many pathogens, in *T. brucei*, the highly repetitive nature and heterozygosity of the antigen-gene arrays had precluded a complete genome assembly. Recently, through a combination of PacBio single-molecule real-time sequencing technology and Hi-C, the haplotype-specific assembly and scaffolding of the long antigen-gene arrays has been successful (Muller *et al*., 2018). This refined genome assembly has been proven critical to perform further analyses on chromatin organization and gene expression, especially regarding *VSG* genes. Among several downstream analyses, which largely benefitted from a refined genome assembly, is Hi-C.

Hi-C and other chromosome conformational capture techniques are a set of powerful molecular biology methods based on proximity labelling, which enable the analysis of chromatin spatial organization. These methods quantify the interaction frequency between genomic loci that are nearby in the 3D nuclear space, but may be far in the linear genome, allowing the identification of enhancer–promoter contacts or chromatin loops for instance (reviewed by Kempfer and Pombo, 2020). Hi-C studies in *T. brucei* have identified key architectural proteins and that a specific chromatin configuration is critical to fine-tune recombination events; indeed, perturbation of that specific architecture triggers switches in antigen expression (Muller *et al*., 2018). Further, virtual 4C analyses survey the interaction frequencies between a bait locus of interest and any other loci in the genome. In *T. brucei*, such analyses have demonstrated that the active-*VSG* ES (but not the silent) as well as genes encoding for other highly abundant proteins interact with the *SL*-array, uncovering a potential enhancer-like mechanism (Faria *et al*., 2020).

Next-generation sequencing techniques including RNA-Seq (transcript abundance), ChIP-Seq (chromatin-association) and CLIP-Seq (RNA-binding) have now been amply used in trypanosomes and other Trypanosomatids. More recently, ATAC-Seq (chromatin accessibility) and single-cell RNA-Seq have been performed in *T. brucei* (Muller *et al*., 2018). The latter opens unprecedented opportunities to investigate differential gene expression during developmental transitions and inherent single-cell variability within a particular life cycle stage.

Huge improvements have been made to imaging techniques and the fast pace of development is truly remarkable. In *T. brucei*, protein and DNA loci have been recently tracked at high resolution, using confocal-based or structured illumination microscopy (XY resolution 100–120 nm), which has been critical to characterize specific sub-nuclear compartments (Glover *et al*., 2016; Budzak *et al*., 2019; Faria *et al*., 2019, 2020). But there is a growing need for methods that can image chromosomes with greater genomic and optical resolution; super resolution microscopy can now allow an XY resolution as low as 20–30 nm. To understand how the genome functions and regulates several key biological processes, it is necessary to visualize many genomic regions simultaneously, not just a few. Recently, there have been huge breakthroughs in other systems, such as OligoFISSEQ, a combination of three methods that employ fluorescence *in situ* sequencing (FISSEQ) of barcoded Oligopaint probes to enable the rapid visualization of multiple targeted genomic regions (Nguyen *et al*., 2020). Another powerful technique is electron cryotomography, an imaging technique used to produce high-resolution 3D views of samples, typically biological macromolecules and cells. In trypanosomatids, it has been used to study flagellar and mitochondrial structures but to my knowledge, not to study supramolecular sub-nuclear complexes. For instance in humans, it was extensively used to study the human NPC (reviewed by Lin and Hoelz, 2019).

The clustered regularly interspaced short palindromic repeats (CRISPR)/CRISPR-associated system (Cas) technology has revolutionized molecular biology; indeed, it is a powerful tool that allows highly efficient and reproducible manipulation of genomic sequences for both locus-specific and genome-wide approaches. But its huge potential is not exclusively linked to the site-directed nuclease activity. A catalytically inactive Cas9 (dCas9) can be used as a universal recruitment platform in order to control transcription, visualize DNA sequences or investigate *in situ* proteomes (Anton *et al*., 2018; Martens *et al*., 2019). Indeed, for the identification of locus-associated proteins, dCas9 can be fused to a FLAG-tag and targeted to a locus of interest; chromatin is then crosslinked and fragmented; dCas9-bound chromatin fragments are subsequently isolated by FLAG-specific antibodies and analysed *via* mass spectrometry (enChIP) (Anton *et al*., 2018). Unlike enChIP, CasID requires the expression of dCas9 fused to the promiscuous biotin ligase BirA*. After the culture medium has been supplemented with exogenous biotin, BirA* catalyses the addition of biotin to lysine residues of proteins that are in close proximity to the dCas9-BirA* fusion protein. Lysis of the cells and denaturation of proteins is then followed by affinity purification of biotinylated peptides, which are identified *via* tandem mass spectrometry (Anton *et al*., 2018; Trinkle-Mulcahy, 2019). Indeed, this DNA-centric system can be used to pull-down proteins that associate with a specific locus; taking the *VSG*-ESs as an example, this system could help identifying factors specifically associated with the active or silent-ESs and factors involved in gene activation or gene silencing.

Additionally, several different dCas9-based systems have been developed to perform programmable control of spatial genome organization, among those is the CRISPR-genome organization (CRISPR-GO) system. It delivers a highly efficient and versatile control over the spatial positioning of genomic loci relative to specific nuclear compartments, including the nuclear periphery, CBs and promyelocytic leukaemia bodies to study how nuclear structure affects gene regulation and cellular function (Wang *et al*., 2018). For example, in *T. brucei*, this could be used to bring genomic loci in proximity to the *SL*-array or the NL and assess how that impacts gene expression. Recently, a CasDrop system was designed to study the formation of phase-separated compartments in the nucleus by enabling liquid condensation of transcriptional regulators at target loci (Shin *et al*., 2018). For example, in *T. brucei*, this could be used to investigate the formation of VEX2 protein condensates at the active *VSG*-ES. CRISPR/Cas9 technology has been successfully adapted to trypanosomes (reviewed by Bryant *et al*., 2019) and proven highly versatile; it will be interesting to see the future developments.

In summary, huge technological advances have been accomplished in the recent years and certainly many more will in the near future. This burst of technological breakthroughs will hopefully pave the way for future discoveries on nuclear and genome organization as well as gene expression control in African trypanosomes and related organisms.

## Concluding remarks

*Trypanosoma brucei* nuclear organization and gene expression present several striking differences when compared to more complex eukaryotes. Multiple lines of evidence strongly support that its monogenic antigen transcription, which is critical for successful antigenic variation, is enforced and facilitated by a key nuclear architecture that involves specific inter-chromosomal interactions and compartmentalization (possibly also modification) of specific factors.

The molecular understanding of the mechanisms underpinning gene expression control in different developmental stages of these parasites is of great importance, as it might aid future vaccine and drug development efforts. For instance, acoziborole, a single-dose oral drug to treat trypanosomiasis, was shown to target cleavage and polyadenylation specificity factor 3 (Wall *et al*., 2018). Therefore, RNA processing is now established as a clinically validated drug target in the African trypanosome. Understanding the context within which drugs work can greatly facilitate the drug discovery process.

Notably, recent technological advances on sequencing, imaging and affinity purification techniques have led to important discoveries and paved the way to novel research avenues regarding nuclear organization and gene expression control in trypanosomes. Indeed, we live in exciting times where the pace of technology development is phenomenal and hopefully will allow us to address long-standing questions in infection biology that were previously inaccessible.

## References

[ref2] Adl SM, Bass D, Lane CE, Lukeš J, Schoch CL, Smirnov A, Agatha S, Berney C, Brown MW, Burki F, Cárdenas P, Čepička I, Chistyakova L, Campo J, Dunthorn M, Edvardsen B, Eglit Y, Guillou L, Hampl V, Heiss AA, Hoppenrath M, James TY, Karnkowska A, Karpov S, Kim E, Kolisko M, Kudryavtsev A, Lahr DJG, Lara E, Le Gall L, Lynn DH, Mann DG, Massana R, Mitchell EAD, Morrow C, Park JS, Pawlowski JW, Powell MJ, Richter DJ, Rueckert S, Shadwick L, Shimano S, Spiegel FW, Torruella G, Youssef N, Zlatogursky V and Zhang Q (2019) Revisions to the classification, nomenclature, and diversity of eukaryotes. Journal of Eukaryotic Microbiology 66, 4–119. 10.1111/jeu.12691 PMC6492006 30257078

[ref3] Alsford S and Horn D (2011) Elongator protein 3b negatively regulates ribosomal DNA transcription in African trypanosomes. Molecular Cellular Biology 31, 1822–1832. 21357738 10.1128/MCB.01026-10 PMC3133226

[ref4] Alsford S and Horn D (2012) Cell-cycle-regulated control of VSG expression site silencing by histones and histone chaperones ASF1A and CAF-1b in *Trypanosoma brucei*. Nucleic Acids Research 40, 10150–10160. 22941664 10.1093/nar/gks813 PMC3488249

[ref5] Alsford NS, Navarro M, Jamnadass HR, Dunbar H, Ackroyd M, Murphy NB, Gull K and Ersfeld K (2003) The identification of circular extrachromosomal DNA in the nuclear genome of *Trypanosoma brucei*. Molecular Microbiology 47, 277–289. 12519183 10.1046/j.1365-2958.2003.03266.x

[ref6] Alsford S, Kawahara T, Isamah C and Horn D (2007) A sirtuin in the African trypanosome is involved in both DNA repair and telomeric gene silencing but is not required for antigenic variation. Molecular Microbiology 63, 724–736. 17214740 10.1111/j.1365-2958.2006.05553.x

[ref7] Alsford S, Turner DJ, Obado SO, Sanchez-Flores A, Glover L, Berriman M, Hertz-Fowler C and Horn D (2011) High-throughput phenotyping using parallel sequencing of RNA interference targets in the African trypanosome. Genome Research 21, 915–924. 21363968 10.1101/gr.115089.110 PMC3106324

[ref8] Ambrósio DL, Lee JH, Panigrahi AK, Nguyen TN, Cicarelli RM and Günzl A (2009) Spliceosomal proteomics in *Trypanosoma brucei* reveal new RNA splicing factors. Eukaryotic Cell 8, 990–1000. 19429779 10.1128/EC.00075-09 PMC2708463

[ref9] Ambrósio DL, Badjatia N and Günzl A (2015) The spliceosomal PRP19 complex of trypanosomes. Molecular Microbiology 95, 885–901. 25524563 10.1111/mmi.12910 PMC4374492

[ref10] Amit-Avraham I, Pozner G, Eshar S, Fastman Y, Kolevzon N, Yavin E and Dzikowski R (2015) Antisense long noncoding RNAs regulate var gene activation in the malaria parasite Plasmodium falciparum. Proceedings of the National Academy of Sciences USA 112, E982–E991. 10.1073/pnas.1420855112 PMC4352787 25691743

[ref11] Anton T, Karg E and Bultmann S (2018) Applications of the CRISPR/Cas system beyond gene editing. Biology Methods and Protocols 3, bpy002. 32161796 10.1093/biomethods/bpy002 PMC6994046

[ref12] Aresta-Branco F, Sanches-Vaz M, Bento F, Rodrigues JA and Figueiredo LM (2019) African trypanosomes expressing multiple VSGs are rapidly eliminated by the host immune system. Proceedings of the National Academy of Sciences USA 116, 20725–20735. 10.1073/pnas.1905120116 PMC6789922 31554700

[ref13] Badjatia N, Ambrósio DL, Lee JH and Günzl A (2013) Trypanosome cdc2-related kinase 9 controls spliced leader RNA cap4 methylation and phosphorylation of RNA polymerase II subunit RPB1. Molecular Cellular Biology 33, 1965–1975. 23478263 10.1128/MCB.00156-13 PMC3647971

[ref14] Bangs JD, Crain PF, Hashizume T, McCloskey JA and Boothroyd JC (1992) Mass spectrometry of mRNA cap 4 from trypanosomatids reveals two novel nucleosides. Journal of Biological Chemistry 267, 9805–9815. 1349605

[ref15] Barquilla A, Crespo JL and Navarro M (2008) Rapamycin inhibits trypanosome cell growth by preventing TOR complex 2 formation. Proceedings of the National Academy of Sciences USA 105, 14579–14584. 10.1073/pnas.0802668105 PMC2567229 18796613

[ref16] Belli SI (2000) Chromatin remodelling during the life cycle of trypanosomatids. International Journal of Parasitology 30, 679–687. 10856501 10.1016/s0020-7519(00)00052-7

[ref17] Berriman M, (2005) The genome of the African trypanosome *Trypanosoma brucei*. Science (New York, N.Y.) 309, 416–422. 10.1126/science.1112642 16020726

[ref18] Brancucci NMB, Bertschi NL, Zhu L, Niederwieser I, Chin WH, Wampfler R, Freymond C, Rottmann M, Felger I, Bozdech Z and Voss TS (2014) Heterochromatin protein 1 secures survival and transmission of malaria parasites. Cell Host & Microbe 16, 165–176. 25121746 10.1016/j.chom.2014.07.004

[ref19] Brandenburg J, Schimanski B, Nogoceke E, Nguyen TN, Padovan JC, Chait BT, Cross GA and Günzl A (2007) Multifunctional class I transcription in *Trypanosoma brucei* depends on a novel protein complex. Embo Journal 26, 4856–4866. 10.1038/sj.emboj.7601905 PMC2099468 17972917

[ref20] Briggs E, Crouch K, Lemgruber L, Lapsley C and McCulloch R (2018) Ribonuclease H1-targeted R-loops in surface antigen gene expression sites can direct trypanosome immune evasion. PLoS Genetics 14, e1007729. 30543624 10.1371/journal.pgen.1007729 PMC6292569

[ref21] Briggs E, Crouch K, Lemgruber L, Hamilton G, Lapsley C and McCulloch R (2019) *Trypanosoma brucei* ribonuclease H2A is an essential R-loop processing enzyme whose loss causes DNA damage during transcription initiation and antigenic variation. Nucleic Acids Research 47, 9180–9197. 31350892 10.1093/nar/gkz644 PMC6753483

[ref22] Bryant JM, Baumgarten S, Glover L, Hutchinson S and Rachidi N (2019) CRISPR in parasitology: not exactly cut and dried!. Trends in Parasitology 35, 409–422. 31006600 10.1016/j.pt.2019.03.004

[ref23] Budzak J, Kerry LE, Aristodemou A, Hall BS, Witmer K, Kushwaha M, Davies C, Povelones ML, McDonald JR, Sur A, Myler PJ and Rudenko G (2019) Dynamic colocalization of 2 simultaneously active *VSG* Expression sites within a single expression-site body in *Trypanosoma brucei*. Proceedings of the National Academy of Sciences of the USA 116, 16561–16570. 31358644 10.1073/pnas.1905552116 PMC6697882

[ref24] Bunnik EM, Cook KB, Varoquaux N, Batugedara G, Prudhomme J, Cort A, Shi L, Andolina C, Ross LS, Brady D, Fidock DA, Nosten F, Tewari R, Sinnis P, Ay F, Vert JP, Noble WS and Le Roch KG (2018) Changes in genome organization of parasite-specific gene families during the Plasmodium transmission stages. Nature Communications 9, 1910. 10.1038/s41467-018-04295-5 PMC5954139 29765020

[ref25] Capewell P, Cren-Travaillé C, Marchesi F, Johnston P, Clucas C, Benson RA, Gorman TA, Calvo-Alvarez E, Crouzols A, Jouvion G, Jamonneau V, Weir W, Stevenson ML, O'Neill K, Cooper A, Swar NK, Bucheton B, Ngoyi DM, Garside P, Rotureau B and MacLeod A (2016) The skin is a significant but overlooked anatomical reservoir for vector-borne African trypanosomes. Elife 5, e17716. 10.7554/eLife.17716 PMC5065312 27653219

[ref26] Cestari I and Stuart K (2015) Inositol phosphate pathway controls transcription of telomeric expression sites in trypanosomes. Proceedings of the National Academy of Sciences of the USA 112, E2803–E2812. 25964327 10.1073/pnas.1501206112 PMC4450425

[ref27] Cestari I and Stuart K (2018) Transcriptional regulation of telomeric expression sites and antigenic variation in trypanosomes. Current Genomics 19, 119–132. 29491740 10.2174/1389202918666170911161831 PMC5814960

[ref28] Cestari I, McLeland-Wieser H and Stuart K (2019) Nuclear phosphatidylinositol 5-phosphatase is essential for allelic exclusion of variant surface glycoprotein genes in trypanosomes. Molecular Cellular Biology 39, 3. 10.1128/MCB.00395-18 PMC6336139 30420356

[ref29] Chaves I, Zomerdijk J, Dirks-Mulder A, Dirks RW, Raap AK and Borst P (1998) Subnuclear localization of the active variant surface glycoprotein gene expression site in *Trypanosoma brucei*. Proceedings of the National Academy of Sciences of the USA 95, 12328–12333. 9770486 10.1073/pnas.95.21.12328 PMC22831

[ref30] Chaves I, Rudenko G, Dirks-Mulder A, Cross M and Borst P (1999) Control of variant surface glycoprotein gene-expression sites in *Trypanosoma brucei*. Embo Journal 18, 4846–4855. 10.1093/emboj/18.17.4846 PMC1171556 10469662

[ref31] Clayton C (2019) Regulation of gene expression in trypanosomatids: living with polycistronic transcription. Open Biology 9, 190072. 31164043 10.1098/rsob.190072 PMC6597758

[ref32] Cong YS, Wright WE and Shay JW (2002) Human telomerase and its regulation. Microbiology and Molecular Biology Reviews 66, 407–425, table of contents. 12208997 10.1128/MMBR.66.3.407-425.2002 PMC120798

[ref33] Daniels JP, Gull K and Wickstead B (2010) Cell biology of the trypanosome genome. Microbiology and Molecular Biology Reviews 74, 552–569. 21119017 10.1128/MMBR.00024-10 PMC3008170

[ref34] Das A, Zhang Q, Palenchar JB, Chatterjee B, Cross GA and Bellofatto V (2005) Trypanosomal TBP functions with the multisubunit transcription factor tSNAP to direct spliced-leader RNA gene expression. Molecular and Cellular Biology 25, 7314–7322. 16055739 10.1128/MCB.25.16.7314-7322.2005 PMC1190245

[ref35] Das A, Li H, Liu T and Bellofatto V (2006) Biochemical characterization of *Trypanosoma brucei* RNA polymerase II. Molecular Biochemical Parasitology 150, 201–210. 16962183 10.1016/j.molbiopara.2006.08.002

[ref36] Dean S, Sunter JD and Wheeler RJ (2017) Tryptag.org: a trypanosome genome-wide protein localisation resource. Trends in Parasitology 33, 80–82. 27863903 10.1016/j.pt.2016.10.009 PMC5270239

[ref37] Dechat T, Pfleghaar K, Sengupta K, Shimi T, Shumaker DK, Solimando L and Goldman RD (2008) Nuclear lamins: major factors in the structural organization and function of the nucleus and chromatin. Genes and Development 22, 832–853. 18381888 10.1101/gad.1652708 PMC2732390

[ref38] DeGrasse JA, DuBois KN, Devos D, Siegel TN, Sali A, Field MC, Rout MP and Chait BT (2009) Evidence for a shared nuclear pore complex architecture that is conserved from the last common eukaryotic ancestor. Molecular & Cellular Proteomics 8, 2119–2130. 19525551 10.1074/mcp.M900038-MCP200 PMC2742445

[ref39] de Lange T (2005) Shelterin: the protein complex that shapes and safeguards human telomeres. Genes & Development 19, 2100–2110. 16166375 10.1101/gad.1346005

[ref40] Denninger V and Rudenko G (2014) FACT plays a major role in histone dynamics affecting VSG expression site control in *Trypanosoma brucei*. Molecular Microbiology 94, 945–962. 25266856 10.1111/mmi.12812 PMC4625058

[ref41] Devaux S, Lecordier L, Uzureau P, Walgraffe D, Dierick JF, Poelvoorde P, Pays E and Vanhamme L (2006) Characterization of RNA polymerase II subunits of *Trypanosoma brucei*. Molecular Biochemical Parasitology 148, 60–68. 16621069 10.1016/j.molbiopara.2006.02.020

[ref42] Devaux S, Kelly S, Lecordier L, Wickstead B, Perez-Morga D, Pays E, Vanhamme L and Gull K (2007) Diversification of function by different isoforms of conventionally shared RNA polymerase subunits. Molecular Biology of the Cell 18, 1293–1301. 17267688 10.1091/mbc.E06-09-0841 PMC1838988

[ref43] Djikeng A, Shi H, Tschudi C and Ullu E (2001) RNA interference in *Trypanosoma brucei*: cloning of small interfering RNAs provides evidence for retroposon-derived 24–26-nucleotide RNAs. RNA 7, 1522–1530. 11720282 PMC1370195

[ref44] do Nascimento LM, Egler F, Arnold K, Papavisiliou N, Clayton C and Erben E (2020) The trypanosome variant surface glycoprotein mRNA is stabilized by an essential unconventional RNA-binding protein. bioRxiv, 2020.2010.2008.331769.

[ref45] Dossin Fde M and Schenkman S (2005) Actively transcribing RNA polymerase II concentrates on spliced leader genes in the nucleus of *Trypanosoma cruzi*. Eukaryotic Cell 4, 960–970. 15879530 10.1128/EC.4.5.960-970.2005 PMC1140094

[ref46] Dreesen O, Li B and Cross GA (2005) Telomere structure and shortening in telomerase-deficient *Trypanosoma brucei*. Nucleic Acids Research 33, 4536–4543. 16091631 10.1093/nar/gki769 PMC1184224

[ref47] DuBois KN, Alsford S, Holden JM, Buisson J, Swiderski M, Bart JM, Ratushny AV, Wan Y, Bastin P, Barry JD, Navarro M, Horn D, Aitchison JD, Rout MP and Field MC (2012) NUP-1 is a large coiled-coil nucleoskeletal protein in trypanosomes with lamin-like functions. PLoS Biology 10, e1001287. 22479148 10.1371/journal.pbio.1001287 PMC3313915

[ref48] Duraisingh MT and Horn D (2016) Epigenetic regulation of virulence gene expression in parasitic protozoa. Cell Host & Microbe 19, 629–640. 27173931 10.1016/j.chom.2016.04.020 PMC5061559

[ref49] Duraisingh MT, Voss TS, Marty AJ, Duffy MF, Good RT, Thompson JK, Freitas-Junior LH, Scherf A, Crabb BS and Cowman AF (2005) Heterochromatin silencing and locus repositioning linked to regulation of virulence genes in Plasmodium falciparum. Cell 121, 13–24. 15820675 10.1016/j.cell.2005.01.036

[ref50] Elias MC, Marques-Porto R, Freymüller E and Schenkman S (2001) Transcription rate modulation through the *Trypanosoma cruzi* life cycle occurs in parallel with changes in nuclear organisation. Molecular Biochemical Parasitology 112, 79–90. 11166389 10.1016/s0166-6851(00)00349-2

[ref51] Engel C, Gubbey T, Neyer S, Sainsbury S, Oberthuer C, Baejen C, Bernecky C and Cramer P (2017) Structural basis of RNA polymerase I transcription initiation. Cell 169, 120–131.e122. 28340337 10.1016/j.cell.2017.03.003

[ref52] Evers R, Hammer A, Köck J, Jess W, Borst P, Mémet S and Cornelissen AW (1989) *Trypanosoma brucei* contains two RNA polymerase II largest subunit genes with an altered C-terminal domain. Cell 56, 585–597. 2917367 10.1016/0092-8674(89)90581-3

[ref53] Faria J, Glover L, Hutchinson S, Boehm C, Field MC and Horn D (2019) Monoallelic expression and epigenetic inheritance sustained by a *Trypanosoma brucei* variant surface glycoprotein exclusion complex. Nature Communications 10, 3023. 10.1038/s41467-019-10823-8 PMC6617441 31289266

[ref54] Faria J, Luzak V, Muller LSM, Brink BG, Hutchinson S, Glover L, Horn D and Siegel TN (2020) Spatial integration of transcription and splicing in a dedicated compartment sustains monogenic antigen expression in African trypanosomes. Nature Microbiology, *in press*. doi: 10.1038/s41564-020-00833-4. PMC7610597 33432154

[ref55] Ferreira-Cerca S, Pöll G, Kühn H, Neueder A, Jakob S, Tschochner H and Milkereit P (2007) Analysis of the *in vivo* assembly pathway of eukaryotic 40S ribosomal proteins. Molecular Cell 28, 446–457. 17996708 10.1016/j.molcel.2007.09.029

[ref56] Figueiredo LM, Janzen CJ and Cross GA (2008) A histone methyltransferase modulates antigenic variation in African trypanosomes. PLoS Biology 6, e161. 18597556 10.1371/journal.pbio.0060161 PMC2443197

[ref57] Finn EH and Misteli T (2019) Molecular basis and biological function of variability in spatial genome organization. Science (New York, N.Y.) 365, eaaw9498. 10.1126/science.aaw9498 PMC7421438 31488662

[ref58] Finn EH, Pegoraro G, Brandão HB, Valton AL, Oomen ME, Dekker J, Mirny L and Misteli T (2019) Extensive heterogeneity and intrinsic variation in spatial genome organization. Cell 176, 1502–1515.e1510. 30799036 10.1016/j.cell.2019.01.020 PMC6408223

[ref59] Foster HA and Bridger JM (2005) The genome and the nucleus: a marriage made by evolution. Genome organisation and nuclear architecture. Chromosoma 114, 212–229. 16133352 10.1007/s00412-005-0016-6

[ref60] Freitas-Junior LH, Hernandez-Rivas R, Ralph SA, Montiel-Condado D, Ruvalcaba-Salazar OK, Rojas-Meza AP, Mâncio-Silva L, Leal-Silvestre RJ, Gontijo AM, Shorte S and Scherf A (2005) Telomeric heterochromatin propagation and histone acetylation control mutually exclusive expression of antigenic variation genes in malaria parasites. Cell 121, 25–36. 15820676 10.1016/j.cell.2005.01.037

[ref61] Galganski L, Urbanek MO and Krzyzosiak WJ (2017) Nuclear speckles: molecular organization, biological function and role in disease. Nucleic Acids Research 45, 10350–10368. 28977640 10.1093/nar/gkx759 PMC5737799

[ref62] Gibcus JH and Dekker J (2013) The hierarchy of the 3D genome. Molecular Cell 49, 773–782. 23473598 10.1016/j.molcel.2013.02.011 PMC3741673

[ref63] Glover L, Alsford S and Horn D (2013) DNA Break site at fragile subtelomeres determines probability and mechanism of antigenic variation in African trypanosomes. PLoS Pathogens 9, e1003260. 23555264 10.1371/journal.ppat.1003260 PMC3610638

[ref64] Glover L, Hutchinson S, Alsford S and Horn D (2016) VEX1 Controls the allelic exclusion required for antigenic variation in trypanosomes. Proceedings of the National Academy of Sciences of the USA 113, 7225–7230. 27226299 10.1073/pnas.1600344113 PMC4932947

[ref65] Günzl A (2010) The pre-mRNA splicing machinery of trypanosomes: complex or simplified? Eukaryotic Cell 9, 1159–1170. 20581293 10.1128/EC.00113-10 PMC2918933

[ref66] Günzl A, Bruderer T, Laufer G, Schimanski B, Tu LC, Chung HM, Lee PT and Lee MG (2003) RNA Polymerase I transcribes procyclin genes and variant surface glycoprotein gene expression sites in *Trypanosoma brucei*. Eukaryotic Cell 2, 542–551. 12796299 10.1128/EC.2.3.542-551.2003 PMC161450

[ref67] Guo YE, Manteiga JC, Henninger JE, Sabari BR, Dall'Agnese A, Hannett NM, Spille JH, Afeyan LK, Zamudio AV, Shrinivas K, Abraham BJ, Boija A, Decker TM, Rimel JK, Fant CB, Lee TI, Cisse II, Sharp PA, Taatjes DJ and Young RA (2019) Pol II phosphorylation regulates a switch between transcriptional and splicing condensates. Nature 572, 543–548. 31391587 10.1038/s41586-019-1464-0 PMC6706314

[ref68] Hahn S (2004) Structure and mechanism of the RNA polymerase II transcription machinery. Nature Structural & Molecular Biology 11, 394–403. 10.1038/nsmb763 PMC1189732 15114340

[ref69] Hashem Y, des Georges A, Fu J, Buss SN, Jossinet F, Jobe A, Zhang Q, Liao HY, Grassucci RA, Bajaj C, Westhof E, Madison-Antenucci S and Frank J (2013) High-resolution cryo-electron microscopy structure of the *Trypanosoma brucei* ribosome. Nature 494, 385–389. 23395961 10.1038/nature11872 PMC3659406

[ref70] Heger P, Marin B, Bartkuhn M, Schierenberg E and Wiehe T (2012) The chromatin insulator CTCF and the emergence of metazoan diversity. Proceedings of the National Academy of Sciences of the USA 109, 17507–17512. 23045651 10.1073/pnas.1111941109 PMC3491479

[ref71] Hernandez-Verdun D, Roussel P, Thiry M, Sirri V and Lafontaine DL (2010) The nucleolus: structure/function relationship in RNA metabolism. Wiley Interdisciplinary Reviews. RNA 1, 415–431. 21956940 10.1002/wrna.39

[ref72] Hertz-Fowler C, Figueiredo LM, Quail MA, Becker M, Jackson A, Bason N, Brooks K, Churcher C, Fahkro S, Goodhead I, Heath P, Kartvelishvili M, Mungall K, Harris D, Hauser H, Sanders M, Saunders D, Seeger K, Sharp S, Taylor JE, Walker D, White B, Young R, Cross GA, Rudenko G, Barry JD, Louis EJ and Berriman M (2008) Telomeric expression sites are highly conserved in *Trypanosoma brucei*. PLoS ONE 3, e3527. 18953401 10.1371/journal.pone.0003527 PMC2567434

[ref73] Hnisz D, Shrinivas K, Young RA, Chakraborty AK and Sharp PA (2017) A phase separation model for transcriptional control. Cell 169, 13–23. 28340338 10.1016/j.cell.2017.02.007 PMC5432200

[ref74] Holden JM, Koreny L, Obado S, Ratushny AV, Chen WM, Chiang JH, Kelly S, Chait BT, Aitchison JD, Rout MP and Field MC (2014) Nuclear pore complex evolution: a trypanosome Mlp analogue functions in chromosomal segregation but lacks transcriptional barrier activity. Molecular Biology of the Cell 25, 1421–1436. 24600046 10.1091/mbc.E13-12-0750 PMC4004592

[ref75] Hondele M, Sachdev R, Heinrich S, Wang J, Vallotton P, Fontoura BMA and Weis K (2019) DEAD-box ATPases are global regulators of phase-separated organelles. Nature 573, 144–148. 31435012 10.1038/s41586-019-1502-y PMC7617057

[ref76] Hughes K, Wand M, Foulston L, Young R, Harley K, Terry S, Ersfeld K and Rudenko G (2007) A novel ISWI is involved in VSG expression site downregulation in African trypanosomes. Embo Journal 26, 2400–2410. 10.1038/sj.emboj.7601678 PMC1864976 17431399

[ref77] Hury A, Goldshmidt H, Tkacz ID and Michaeli S (2009) Trypanosome spliced-leader-associated RNA (SLA1) localization and implications for spliced-leader RNA biogenesis. Eukaryotic Cell 8, 56–68. 19028994 10.1128/EC.00322-08 PMC2620742

[ref78] Isaac C, Yang Y and Meier UT (1998) Nopp140 functions as a molecular link between the nucleolus and the coiled bodies. Journal of Cell Biology 142, 319–329. 10.1083/jcb.142.2.319 PMC2133063 9679133

[ref79] Jehi SE, Li X, Sandhu R, Ye F, Benmerzouga I, Zhang M, Zhao Y and Li B (2014a) Suppression of subtelomeric VSG switching by *Trypanosoma brucei* TRF requires its TTAGGG repeat-binding activity. Nucleic Acids Research 42, 12899–12911. 25313155 10.1093/nar/gku942 PMC4227783

[ref80] Jehi SE, Wu F and Li B (2014b) *Trypanosoma brucei* TIF2 suppresses VSG switching by maintaining subtelomere integrity. Cell Research 24, 870–885. 24810301 10.1038/cr.2014.60 PMC4085761

[ref81] Karpen GH, Schaefer JE and Laird CD (1988) A Drosophila rRNA gene located in euchromatin is active in transcription and nucleolus formation. Genes & Development 2, 1745–1763. 3149250 10.1101/gad.2.12b.1745

[ref82] Kassem A, Pays E and Vanhamme L (2014) Transcription is initiated on silent variant surface glycoprotein expression sites despite monoallelic expression in *Trypanosoma brucei*. Proceedings of the National Academy of Sciences of the USA 111, 8943–8948. 24889641 10.1073/pnas.1404873111 PMC4066500

[ref83] Kelly S, Singleton W, Wickstead B, Ersfeld K and Gull K (2006) Characterization and differential nuclear localization of Nopp140 and a novel Nopp140-like protein in trypanosomes. Eukaryotic Cell 5, 876–879. 16682465 10.1128/EC.5.5.876-879.2006 PMC1459678

[ref84] Kelly S, Kramer S, Schwede A, Maini PK, Gull K and Carrington M (2012) Genome organization is a major component of gene expression control in response to stress and during the cell division cycle in trypanosomes. Open Biology 2, 120033. 22724062 10.1098/rsob.120033 PMC3376733

[ref85] Kempfer R and Pombo A (2020) Methods for mapping 3D chromosome architecture. Nature Reviews Genetics 21, 207–226. 10.1038/s41576-019-0195-2 31848476

[ref86] Kieft R, Zhang Y, Marand AP, Moran JD, Bridger R, Wells L, Schmitz RJ and Sabatini R (2020) Identification of a novel base J binding protein complex involved in RNA polymerase II transcription termination in trypanosomes. PLoS Genetics 16, e1008390. 32084124 10.1371/journal.pgen.1008390 PMC7055916

[ref87] Kim J, Venkata NC, Hernandez Gonzalez GA, Khanna N and Belmont AS (2020) Gene expression amplification by nuclear speckle association. Journal of Cell Biology 219, 1. 10.1083/jcb.201904046 PMC7039209 31757787

[ref88] Kornblihtt AR, de la Mata M, Fededa JP, Munoz MJ and Nogues G (2004) Multiple links between transcription and splicing. RNA 10, 1489–1498. 15383674 10.1261/rna.7100104 PMC1370635

[ref89] Koumandou VL, Natesan SK, Sergeenko T and Field MC (2008) The trypanosome transcriptome is remodelled during differentiation but displays limited responsiveness within life stages. BMC Genomics 9, 298. 18573209 10.1186/1471-2164-9-298 PMC2443814

[ref90] Landeira D and Navarro M (2007) Nuclear repositioning of the *VSG* Promoter during developmental silencing in *Trypanosoma brucei*. The Journal of Cell Biology 176, 133–139. 17210949 10.1083/jcb.200607174 PMC2063932

[ref91] Landeira D, Bart JM, Van Tyne D and Navarro M (2009) Cohesin regulates *VSG* monoallelic expression in trypanosomes. The Journal of Cell Biology 186, 243–254. 19635842 10.1083/jcb.200902119 PMC2717648

[ref92] Lee JH, Nguyen TN, Schimanski B and Günzl A (2007) Spliced leader RNA gene transcription in *Trypanosoma brucei* requires transcription factor TFIIH. Eukaryotic Cell 6, 641–649. 17259543 10.1128/EC.00411-06 PMC1865645

[ref93] Lee JH, Cai G, Panigrahi AK, Dunham-Ems S, Nguyen TN, Radolf JD, Asturias FJ and Günzl A (2010) A TFIIH-associated mediator head is a basal factor of small nuclear spliced leader RNA gene transcription in early-diverged trypanosomes. Molecular and Cellular Biology 30, 5502–5513. 20876299 10.1128/MCB.00966-10 PMC2976424

[ref94] Lemieux JE, Kyes SA, Otto TD, Feller AI, Eastman RT, Pinches RA, Berriman M, Su XZ and Newbold CI (2013) Genome-wide profiling of chromosome interactions in *Plasmodium falciparum* characterizes nuclear architecture and reconfigurations associated with antigenic variation. Molecular Microbiology 90, 519–537. 23980881 10.1111/mmi.12381 PMC3894959

[ref95] Liang XH, Liu Q, Liu L, Tschudi C and Michaeli S (2006) Analysis of spliceosomal complexes in *Trypanosoma brucei* and silencing of two splicing factors Prp31 and Prp43. Molecular Biochemical Parasitology 145, 29–39. 16219373 10.1016/j.molbiopara.2005.09.004

[ref96] Lin DH and Hoelz A (2019) The structure of the nuclear pore complex (an update). Annual Review of Biochemistry 88, 725–783. 10.1146/annurev-biochem-062917-011901 PMC6588426 30883195

[ref97] Liu Z, Gutierrez-Vargas C, Wei J, Grassucci RA, Ramesh M, Espina N, Sun M, Tutuncuoglu B, Madison-Antenucci S, Woolford JL Jr, Tong L and Frank J (2016) Structure and assembly model for the *Trypanosoma cruzi* 60S ribosomal subunit. Proceedings of the National Academy of Sciences of the USA 113, 12174–12179. 27791004 10.1073/pnas.1614594113 PMC5087005

[ref98] López-Farfán D, Bart JM, Rojas-Barros DI and Navarro M (2014) SUMOylation by the E3 ligase TbSIZ1/PIAS1 positively regulates VSG expression in *Trypanosoma brucei*. PLoS Pathogens 10, e1004545. 25474309 10.1371/journal.ppat.1004545 PMC4256477

[ref99] Machyna M, Neugebauer KM and Staněk D (2015) Coilin: the first 25 years. RNA Biology 12, 590–596. 25970135 10.1080/15476286.2015.1034923 PMC4615369

[ref100] Maishman L, Obado SO, Alsford S, Bart JM, Chen WM, Ratushny AV, Navarro M, Horn D, Aitchison JD, Chait BT, Rout MP and Field MC (2016) Co-dependence between trypanosome nuclear lamina components in nuclear stability and control of gene expression. Nucleic Acids Research 44, 10554–10570. 27625397 10.1093/nar/gkw751 PMC5159534

[ref101] Martens KJA, van Beljouw SPB, van der Els S, Vink JNA, Baas S, Vogelaar GA, Brouns SJJ, van Baarlen P, Kleerebezem M and Hohlbein J (2019) Visualisation of dCas9 target search *in vivo* using an open-microscopy framework. Nature Communications 10, 3552. 10.1038/s41467-019-11514-0 PMC6685946 31391532

[ref102] Martínez-Calvillo S, Florencio-Martínez LE and Nepomuceno-Mejía T (2019) Nucleolar structure and function in trypanosomatid protozoa. Cells 8, 421. 10.3390/cells8050421 PMC6562600 31071985

[ref103] Merkenschlager M and Odom DT (2013) CTCF and cohesin: linking gene regulatory elements with their targets. Cell 152, 1285–1297. 23498937 10.1016/j.cell.2013.02.029

[ref104] Michaeli S (2011) Trans-splicing in trypanosomes: machinery and its impact on the parasite transcriptome. Future Microbiology 6, 459–474. 21526946 10.2217/fmb.11.20

[ref105] Millau JF and Gaudreau L (2011) CTCF, cohesin, and histone variants: connecting the genome. Biochemical Cellular Biology 89, 505–513. 10.1139/o11-052 21970734

[ref106] Misteli T (2007) Beyond the sequence: cellular organization of genome function. Cell 128, 787–800. 17320514 10.1016/j.cell.2007.01.028

[ref107] Monahan K and Lomvardas S (2015) Monoallelic expression of olfactory receptors. Annual Review of Cell and Developmental Biology 31, 721–740. 10.1146/annurev-cellbio-100814-125308 PMC4882762 26359778

[ref108] Monahan K, Horta A and Lomvardas S (2019) LHX2- and LDB1-mediated trans interactions regulate olfactory receptor choice. Nature 565, 448–453. 30626972 10.1038/s41586-018-0845-0 PMC6436840

[ref109] Muller LSM, Cosentino RO, Forstner KU, Guizetti J, Wedel C, Kaplan N, Janzen CJ, Arampatzi P, Vogel J, Steinbiss S, Otto TD, Saliba AE, Sebra RP and Siegel TN (2018) Genome organization and DNA accessibility control antigenic variation in trypanosomes. Nature 563, 121–125. 30333624 10.1038/s41586-018-0619-8 PMC6784898

[ref110] Nanavaty V, Sandhu R, Jehi SE, Pandya UM and Li B (2017) *Trypanosoma brucei* RAP1 maintains telomere and subtelomere integrity by suppressing TERRA and telomeric RNA:DNA hybrids. Nucleic Acids Research 45, 5785–5796. 28334836 10.1093/nar/gkx184 PMC5449629

[ref111] Narayanan MS and Rudenko G (2013) TDP1 Is an HMG chromatin protein facilitating RNA polymerase I transcription in African trypanosomes. Nucleic Acids Research 41, 2981–2992. 23361461 10.1093/nar/gks1469 PMC3597664

[ref112] Navarro M and Cross GA (1996) DNA rearrangements associated with multiple consecutive directed antigenic switches in *Trypanosoma brucei*. Molecular Cellular Biology 16, 3615–3625. 8668178 10.1128/mcb.16.7.3615 PMC231357

[ref113] Navarro M and Gull K (2001) A pol I transcriptional body associated with *VSG* mono-allelic expression in *Trypanosoma brucei*. Nature 414, 759–763. 11742402 10.1038/414759a

[ref114] Navarro M, Peñate X and Landeira D (2007) Nuclear architecture underlying gene expression in *Trypanosoma brucei*. Trends in Microbiology 15, 263–270. 17481901 10.1016/j.tim.2007.04.004

[ref115] Nepomuceno-Mejía T, Lara-Martínez R, Cevallos AM, López-Villaseñor I, Jiménez-García LF and Hernández R (2010) The *Trypanosoma cruzi* nucleolus: a morphometrical analysis of cultured epimastigotes in the exponential and stationary phases. FEMS Microbiology Letters 313, 41–46. 20880201 10.1111/j.1574-6968.2010.02117.x

[ref116] Nguyen TN, Schimanski B, Zahn A, Klumpp B and Günzl A (2006) Purification of an eight subunit RNA polymerase I complex in *Trypanosoma brucei*. Molecular Biochemical Parasitology 149, 27–37. 16730080 10.1016/j.molbiopara.2006.02.023

[ref117] Nguyen TN, Schimanski B and Günzl A (2007) Active RNA polymerase I of *Trypanosoma brucei* harbors a novel subunit essential for transcription. Molecular Cellular Biology 27, 6254–6263. 17606628 10.1128/MCB.00382-07 PMC1952147

[ref118] Nguyen TN, Nguyen BN, Lee JH, Panigrahi AK and Günzl A (2012) Characterization of a novel class I transcription factor A (CITFA) subunit that is indispensable for transcription by the multifunctional RNA polymerase I of *Trypanosoma brucei*. Eukaryotic Cell 11, 1573–1581. 23104567 10.1128/EC.00250-12 PMC3536272

[ref119] Nguyen HQ, Chattoraj S, Castillo D, Nguyen SC, Nir G, Lioutas A, Hershberg EA, Martins NMC, Reginato PL, Hannan M, Beliveau BJ, Church GM, Daugharthy ER, Marti-Renom MA and Wu CT (2020) 3D Mapping and accelerated super-resolution imaging of the human genome using *in situ* sequencing. Nature Methods 17, 822–832. 32719531 10.1038/s41592-020-0890-0 PMC7537785

[ref120] Obado SO, Brillantes M, Uryu K, Zhang W, Ketaren NE, Chait BT, Field MC and Rout MP (2016) Interactome mapping reveals the evolutionary history of the nuclear pore complex. PLoS Biology 14, e1002365. 26891179 10.1371/journal.pbio.1002365 PMC4758718

[ref121] Ogbadoyi E, Ersfeld K, Robinson D, Sherwin T and Gull K (2000) Architecture of the *Trypanosoma brucei* nucleus during interphase and mitosis. Chromosoma 108, 501–513. 10794572 10.1007/s004120050402

[ref122] Ori A, Banterle N, Iskar M, Andrés-Pons A, Escher C, Khanh Bui H, Sparks L, Solis-Mezarino V, Rinner O, Bork P, Lemke EA and Beck M (2013) Cell type-specific nuclear pores: a case in point for context-dependent stoichiometry of molecular machines. Molecular Systems Biology 9, 648. 23511206 10.1038/msb.2013.4 PMC3619942

[ref123] Ottaviani A, Gilson E and Magdinier F (2008) Telomeric position effect: from the yeast paradigm to human pathologies? Biochimie 90, 93–107. 17868970 10.1016/j.biochi.2007.07.022

[ref124] Otto TD, Böhme U, Sanders M, Reid A, Bruske EI, Duffy CW, Bull PC, Pearson RD, Abdi A, Dimonte S, Stewart LB, Campino S, Kekre M, Hamilton WL, Claessens A, Volkman SK, Ndiaye D, Amambua-Ngwa A, Diakite M, Fairhurst RM, Conway DJ, Franck M, Newbold CI and Berriman M (2018) Long read assemblies of geographically dispersed *Plasmodium falciparum* isolates reveal highly structured subtelomeres. Wellcome Open Research 3, 52. 29862326 10.12688/wellcomeopenres.14571.1 PMC5964635

[ref125] Outters P, Jaeger S, Zaarour N and Ferrier P (2015) Long-range control of V(D)J recombination & allelic exclusion: modeling views. Advances in Immunology 128, 363–413. 26477371 10.1016/bs.ai.2015.08.002

[ref126] Palfi Z, Lücke S, Lahm HW, Lane WS, Kruft V, Bragado-Nilsson E, Séraphin B and Bindereif A (2000) The spliceosomal snRNP core complex of *Trypanosoma brucei*: cloning and functional analysis reveals seven Sm protein constituents. Proceedings of the National Academy of Sciences of the USA 97, 8967–8972. 10900267 10.1073/pnas.150236097 PMC16805

[ref127] Povelones ML, Gluenz E, Dembek M, Gull K and Rudenko G (2012) Histone H1 plays a role in heterochromatin formation and VSG expression site silencing in *Trypanosoma brucei*. PLoS Pathogens 8, e1003010. 23133390 10.1371/journal.ppat.1003010 PMC3486875

[ref128] Preusser C, Palfi Z and Bindereif A (2009) Special Sm core complex functions in assembly of the U2 small nuclear ribonucleoprotein of *Trypanosoma brucei*. Eukaryotic Cell 8, 1228–1234. 19542313 10.1128/EC.00090-09 PMC2725569

[ref129] Prohaska K and Williams N (2009) Assembly of the *Trypanosoma brucei* 60S ribosomal subunit nuclear export complex requires trypanosome-specific proteins P34 and P37. Eukaryotic Cell 8, 77–87. 18723605 10.1128/EC.00234-08 PMC2620753

[ref130] Proudfoot NJ, Furger A and Dye MJ (2002) Integrating mRNA processing with transcription. Cell 108, 501–512. 11909521 10.1016/s0092-8674(02)00617-7

[ref131] Quinodoz SA, Ollikainen N, Tabak B, Palla A, Schmidt JM, Detmar E, Lai MM, Shishkin AA, Bhat P, Takei Y, Trinh V, Aznauryan E, Russell P, Cheng C, Jovanovic M, Chow A, Cai L, McDonel P, Garber M and Guttman M (2018) Higher-order inter-chromosomal hubs shape 3D genome organization in the nucleus. Cell 174, 744–757.e724. 29887377 10.1016/j.cell.2018.05.024 PMC6548320

[ref132] Rao SS, Huntley MH, Durand NC, Stamenova EK, Bochkov ID, Robinson JT, Sanborn AL, Machol I, Omer AD, Lander ES and Aiden EL (2014) A 3D map of the human genome at kilobase resolution reveals principles of chromatin looping. Cell 159, 1665–1680. 25497547 10.1016/j.cell.2014.11.021 PMC5635824

[ref133] Raska I, Shaw PJ and Cmarko D (2006) Structure and function of the nucleolus in the spotlight. Current Opinion in Cell Biology 18, 325–334. 16687244 10.1016/j.ceb.2006.04.008

[ref134] Razin SV and Gavrilov AA (2020) The role of liquid-liquid phase separation in the compartmentalization of cell nucleus and spatial genome organization. Biochemistry (Mosc) 85, 643–650. 32586227 10.1134/S0006297920060012

[ref135] Reis H, Schwebs M, Dietz S, Janzen CJ and Butter F (2018) TelAP1 links telomere complexes with developmental expression site silencing in African trypanosomes. Nucleic Acids Research 46, 2820–2833. 29385523 10.1093/nar/gky028 PMC5888660

[ref136] Reynolds DL, Hofmeister BT, Cliffe L, Siegel TN, Anderson BA, Beverley SM, Schmitz RJ and Sabatini R (2016) Base J represses genes at the end of polycistronic gene clusters in *Leishmania major* by promoting RNAP II termination. Molecular Microbiology 101, 559–574. 27125778 10.1111/mmi.13408 PMC5038137

[ref137] Rico E, Jeacock L, Kovářová J and Horn D (2018) Inducible high-efficiency CRISPR-Cas9-targeted gene editing and precision base editing in African trypanosomes. Scientific Reports 8, 7960. 29785042 10.1038/s41598-018-26303-w PMC5962531

[ref138] Rout MP, Obado SO, Schenkman S and Field MC (2017) Specialising the parasite nucleus: pores, lamins, chromatin, and diversity. PLoS Pathogens 13, e1006170. 28253370 10.1371/journal.ppat.1006170 PMC5333908

[ref139] Rubbi CP and Milner J (2003) Disruption of the nucleolus mediates stabilization of p53 in response to DNA damage and other stresses. Embo Journal 22, 6068–6077. 10.1093/emboj/cdg579 PMC275437 14609953

[ref140] Saha A, Nanavaty VP and Li B (2020) Telomere and subtelomere R-loops and antigenic variation in trypanosomes. Journal of Molecular Biology 432, 4167–4185. 31682833 10.1016/j.jmb.2019.10.025 PMC7195242

[ref141] Sandhu R, Sanford S, Basu S, Park M, Pandya UM, Li B and Chakrabarti K (2013) A trans-spliced telomerase RNA dictates telomere synthesis in *Trypanosoma brucei*. Cell Research 23, 537–551. 23478302 10.1038/cr.2013.35 PMC3616428

[ref142] Sawyer IA, Sturgill D, Sung MH, Hager GL and Dundr M (2016) Cajal body function in genome organization and transcriptome diversity. Bioessays 38, 1197–1208. 27767214 10.1002/bies.201600144 PMC5225948

[ref143] Schimanski B, Nguyen TN and Günzl A (2005) Characterization of a multisubunit transcription factor complex essential for spliced-leader RNA gene transcription in *Trypanosoma brucei*. Molecular Cell Biology 25, 7303–7313. 10.1128/MCB.25.16.7303-7313.2005 PMC1190248 16055738

[ref144] Schimanski B, Brandenburg J, Nguyen TN, Caimano MJ and Günzl A (2006) A TFIIB-like protein is indispensable for spliced leader RNA gene transcription in *Trypanosoma brucei*. Nucleic Acids Research 34, 1676–1684. 16554554 10.1093/nar/gkl090 PMC1409817

[ref145] Schoenfelder S and Fraser P (2019) Long-range enhancer-promoter contacts in gene expression control. Nature Reviews Genetics 20, 437–455. 10.1038/s41576-019-0128-0 31086298

[ref146] Schulz D, Mugnier MR, Paulsen EM, Kim HS, Chung CW, Tough DF, Rioja I, Prinjha RK, Papavasiliou FN and Debler EW (2015) Bromodomain proteins contribute to maintenance of bloodstream form stage identity in the African trypanosome. PLoS Biology 13, e1002316. 26646171 10.1371/journal.pbio.1002316 PMC4672894

[ref147] Schulz D, Zaringhalam M, Papavasiliou FN and Kim HS (2016) Base J and H3.V regulate transcriptional termination in *Trypanosoma brucei*. PLoS Genetics 12, e1005762. 26796638 10.1371/journal.pgen.1005762 PMC4721952

[ref148] Shevelyov YY and Ulianov SV (2019) The nuclear lamina as an organizer of chromosome architecture. Cells 8, 136. 10.3390/cells8020136 PMC6406483 30744037

[ref149] Shin Y, Chang YC, Lee DSW, Berry J, Sanders DW, Ronceray P, Wingreen NS, Haataja M and Brangwynne CP (2018) Liquid nuclear condensates mechanically sense and restructure the genome. Cell 175, 1481–1491.e1413. 30500535 10.1016/j.cell.2018.10.057 PMC6724728

[ref150] Shpargel KB, Ospina JK, Tucker KE, Matera AG and Hebert MD (2003) Control of Cajal body number is mediated by the coilin C-terminus. Journal of Cell Science 116, 303–312. 12482916 10.1242/jcs.00211

[ref151] Spann TP, Moir RD, Goldman AE, Stick R and Goldman RD (1997) Disruption of nuclear lamin organization alters the distribution of replication factors and inhibits DNA synthesis. Journal of Cell Biology 136, 1201–1212. 10.1083/jcb.136.6.1201 PMC2132512 9087437

[ref152] Srivastava A, Badjatia N, Lee JH, Hao B and Günzl A (2018) An RNA polymerase II-associated TFIIF-like complex is indispensable for SL RNA gene transcription in *Trypanosoma brucei*. Nucleic Acids Research 46, 1695–1709. 29186511 10.1093/nar/gkx1198 PMC5829719

[ref153] Strom AR and Brangwynne CP (2019) The liquid nucleome – phase transitions in the nucleus at a glance. Journal of Cell Science 132, jcs235093. 31754043 10.1242/jcs.235093 PMC6899023

[ref154] Tan J and Lan L (2020) The DNA secondary structures at telomeres and genome instability. Cell & Bioscience 10, 47. 32257105 10.1186/s13578-020-00409-z PMC7104500

[ref155] Tkacz ID, Lustig Y, Stern MZ, Biton M, Salmon-Divon M, Das A, Bellofatto V and Michaeli S (2007) Identification of novel snRNA-specific Sm proteins that bind selectively to U2 and U4 snRNAs in *Trypanosoma brucei*. RNA 13, 30–43. 17105994 10.1261/rna.174307 PMC1705756

[ref156] Tkacz ID, Gupta SK, Volkov V, Romano M, Haham T, Tulinski P, Lebenthal I and Michaeli S (2010) Analysis of spliceosomal proteins in Trypanosomatids reveals novel functions in mRNA processing. Journal of Biological Chemistry 285, 27982–27999. 10.1074/jbc.M109.095349 PMC2934664 20592024

[ref157] Tonkin CJ, Carret CK, Duraisingh MT, Voss TS, Ralph SA, Hommel M, Duffy MF, Silva LM, Scherf A, Ivens A, Speed TP, Beeson JG and Cowman AF (2009) Sir2 paralogues cooperate to regulate virulence genes and antigenic variation in *Plasmodium falciparum*. PLoS Biology 7, e84. 19402747 10.1371/journal.pbio.1000084 PMC2672602

[ref158] Trindade S, Rijo-Ferreira F, Carvalho T, Pinto-Neves D, Guegan F, Aresta-Branco F, Bento F, Young SA, Pinto A, Van Den Abbeele J, Ribeiro RM, Dias S, Smith TK and Figueiredo LM (2016) *Trypanosoma brucei* parasites occupy and functionally adapt to the adipose tissue in mice. Cell Host & Microbe 19, 837–848. 27237364 10.1016/j.chom.2016.05.002 PMC4906371

[ref159] Trinkle-Mulcahy L (2019) Recent advances in proximity-based labeling methods for interactome mapping. F1000Research 8. 10.12688/f1000research.16903.1 PMC6357996 30774936

[ref160] Uzureau P, Daniels JP, Walgraffe D, Wickstead B, Pays E, Gull K and Vanhamme L (2008) Identification and characterization of two trypanosome TFIIS proteins exhibiting particular domain architectures and differential nuclear localizations. Molecular Microbiology 69, 1121–1136. 18627464 10.1111/j.1365-2958.2008.06348.x PMC2610381

[ref161] Vanhamme L, Poelvoorde P, Pays A, Tebabi P, Van Xong H and Pays E (2000) Differential RNA elongation controls the variant surface glycoprotein gene expression sites of *Trypanosoma brucei*. Molecular Microbiology 36, 328–340. 10792720 10.1046/j.1365-2958.2000.01844.x

[ref162] Viegas IJ, de Macedo JP, De Niz M, Rodrigues JA, Aresta-Branco F, Jaffrey SR and Figueiredo LM (2020) N6-methyladenosine in poly(A) tails stabilize VSG transcripts. bioRxiv. doi: 10.1101/2020.01.30.925776. PMC9150445 35355019

[ref163] Volz JC, Bártfai R, Petter M, Langer C, Josling GA, Tsuboi T, Schwach F, Baum J, Rayner JC, Stunnenberg HG, Duffy MF and Cowman AF (2012) PfSET10, a *Plasmodium falciparum* methyltransferase, maintains the active var gene in a poised state during parasite division. Cell Host & Microbe 11, 7–18. 22264509 10.1016/j.chom.2011.11.011

[ref164] Walgraffe D, Devaux S, Lecordier L, Dierick JF, Dieu M, Van den Abbeele J, Pays E and Vanhamme L (2005) Characterization of subunits of the RNA polymerase I complex in *Trypanosoma brucei*. Molecular Biochemical Parasitology 139, 249–260. 15664659 10.1016/j.molbiopara.2004.11.014

[ref165] Wall RJ, Rico E, Lukac I, Zuccotto F, Elg S, Gilbert IH, Freund Y, Alley MRK, Field MC, Wyllie S and Horn D (2018) Clinical and veterinary trypanocidal benzoxaboroles target CPSF3. Proceedings of the National Academy of Sciences of the USA 115, 9616–9621. 30185555 10.1073/pnas.1807915115 PMC6156652

[ref166] Wang QP, Kawahara T and Horn D (2010) Histone deacetylases play distinct roles in telomeric VSG expression site silencing in African trypanosomes. Molecular Microbiology 77, 1237–1245. 20624217 10.1111/j.1365-2958.2010.07284.x PMC2941730

[ref167] Wang H, Xu X, Nguyen CM, Liu Y, Gao Y, Lin X, Daley T, Kipniss NH, La Russa M and Qi LS (2018) CRISPR-mediated programmable 3D genome positioning and nuclear organization. Cell 175, 1405–1417.e1414. 30318144 10.1016/j.cell.2018.09.013 PMC6239909

[ref168] Wedel C, Förstner KU, Derr R and Siegel TN (2017) GT-rich promoters can drive RNA pol II transcription and deposition of H2A.Z in African trypanosomes. Embo Journal 36, 2581–2594. 10.15252/embj.201695323 PMC5579346 28701485

[ref169] Wickstead B, Ersfeld K and Gull K (2004) The small chromosomes of *Trypanosoma brucei* involved in antigenic variation are constructed around repetitive palindromes. Genome Research 14, 1014–1024. 15173109 10.1101/gr.2227704 PMC419779

[ref170] Yang X, Figueiredo LM, Espinal A, Okubo E and Li B (2009) RAP1 is essential for silencing telomeric variant surface glycoprotein genes in *Trypanosoma brucei*. Cell 137, 99–109. 19345190 10.1016/j.cell.2009.01.037 PMC2673096

